# Dynamics of starch formation and gene expression during grain filling and its possible influence on grain quality

**DOI:** 10.1038/s41598-024-57010-4

**Published:** 2024-03-21

**Authors:** Sanjeeva Rao Durbha, N. Siromani, V. Jaldhani, T. Krishnakanth, Vishnukiran Thuraga, C. N. Neeraja, D. Subrahmanyam, R. M. Sundaram

**Affiliations:** ICAR-Indian Institute of Rice Research, Rajendranagar, Hyderabad, 500030 India

**Keywords:** Rice, Grain filling, Amylose, Amylopectin, Starch granule, RT-qPCR, BE2b, Plant sciences, Photosynthesis, Plant biotechnology

## Abstract

In rice, grain filling is a crucial stage where asynchronous filling of the pollinated spikelet’s of the panicle occurs. It can influence both grain quality and yield. In rice grain, starch is the dominant component and contains amylose and amylopectin. Amylose content is the chief cooking quality parameter, however, rice varieties having similar amylose content varied in other parameters. Hence, in this study, a set of varieties varying in yield (04) and another set (12) of varieties that are similar in amylose content with variation in gel consistency and alkali spreading value were used. Panicles were collected at various intervals and analysed for individual grain weight and quantities of amylose and amylopectin. Gas exchange parameters were measured in varieties varying in yield. Upper branches of the panicles were collected from rice varieties having similar amylose content and were subjected to gene expression analysis with fourteen gene specific primers of starch synthesis. Results indicate that grain filling was initiated simultaneously in multiple branches. Amylose and amylopectin quantities increased with the increase in individual grain weight. However, the pattern of regression lines of amylose and amylopectin percentages with increase in individual grain weight varied among the varieties. Gas exchange parameters like photosynthetic rate, stomatal conductance, intercellular CO_2_ and transpiration rate decreased with the increase in grain filling period in both good and poor yielding varieties. However, they decreased more in poor yielders. Expression of fourteen genes varied among the varieties and absence of SBE2b can be responsible for medium or soft gel consistency.

## Introduction

Rice (*Oryza sativa L.*) is one of the staple foods and it feeds more than half of the world’s population. In the last fifty years, human population has doubled^1^ and crossed eight billion. This rapid growth in the population demands for more food grain production. In addition to yield, grain quality is also important for the large-scale adoption of a variety^2^.

In rice, grain quality is assessed for milling yield, grain type, chalkiness and cooking quality. Milling yield is the percentage of full or unbroken rice obtained after removing the husk and bran layers^2^. Grain type represents the length to breadth ratio of the grain. Based on the values, rice varieties were classified into long slender (LS), long bold (LB), medium slender (MS), medium bold (MB), short slender (SS) and short bold (SB)^3^. Cooking quality includes aroma, alkali spreading value (ASV), gel consistency (GC) and amylose content (AC)^4–6^. The intensity of the aroma that emanates from the cooked grains of basmati and aromatic short grain rice is examined by a set of trained panel members. Alkali spreading value indirectly represents the gelatinization temperature of the starch granules^4^. Gel consistency gives the tenderness or hardness of the cooked grain^6^. Amylose content is the most important parameter which is useful to select rice varieties having good cooking quality^3,5,7–10^.

Starch is the major component of rice grain followed by proteins, lipids and traces of other compounds (vitamins, minerals, organic acids, structural polysaccharides, etc.). Starch includes two polysaccharides, amylose (linear) and amylopectin (branched). Amylose content ranges from 0% (waxy) to above 30% (high) while the remaining starch is of amylopectin^5^. Generally, the cooked grains of rice having low or waxy (~ 0%) amylose are sticky while the cooked grains of rice having intermediate to high amylose are well separated which are mostly preferred in India^7^. So far, above thousand rice varieties were released in India, the cooking quality of a few varieties like Samba mashuri, Swarna and PB 1121 is more popular among all the stake holders of rice. Apart from the non-scientific reasons in the popularization of rice varieties, it is essential to explore the influence of other areas like grain filling and shelf-life on rice grain quality.

Of these, grain filling can be crucial for both yield and quality. Efficient sink and source are required for efficient grain filling in a rice variety. Sink capacity was enhanced by increasing the available spikelet number in the panicle, however, grains present at the lower branches of the panicle are either unfilled or partially filled^11^ leading to variation in the individual grain weights^12^. Further, superior grains in the middle and upper areas of the panicle could fill better than the inferior grains and it may be due to sucrose transformation and starch synthesis^13–15^. As the grain weight of the individual rice grain increases during grain filling to a maximum value, each grain weight value can represent a growth stage while attaining the maximum weight. Although total starch content was presented per grain^16–18^, 30 grains each from distal primary branches as well as proximal secondary branches of the panicle were collectively grounded to rice powders which were used for the estimation of amylose content^16^. Similarly, top as well as basal spikelet’s collected from seven plants per replicate were used for starch estimation^18^. Hence, to the best of our knowledge, amylose and amylopectin contents are yet to be determined in individual grains during grain filling. Further, the quantities of starch components, amylose and amylopectin, in individual grains at different grain weights present at various branches of the panicle can be more useful to understand grain filling in rice varieties that vary in yield as well as varieties that vary in grain quality.

Rice is a self-pollinating crop and the spikelet remains open for 30–70 min. Like other grass species, in rice, two fertilizations happen- one male nucleus with an egg nucleus forms the embryo and another male nucleus with two polar nuclei forms the triploid endosperm^17^. In the endosperm, initially, nuclei undergo division followed by cellular development and the number of cells will be higher in primary spikelets that undergone anthesis early than the inferior spikelets^19^. The outermost 3–6 cell layers differentiate into aleurone layers while the cells in the sub-aleurone layers become starch storage tissue grow in size upto 30th DAF of grain filling^17^. Small spherical or elliptical amyloplasts will form and they grow in size having polygonal or irregular shaped starch granules of 3–8 µm^20^. Initially, the more amount of hexose than sucrose reaches the kernel, however, the proportion of sucrose increases within a few days ^21^ and water leaves the kernel^22^.

Starch accumulation in the endosperm starts with the breakdown of sucrose by cell wall invertases (CINs) into glucose and fructose^23^ in the apoplasmic space or sucrose also gets transported to cytoplasm of endosperm cells through an ATP dependent sucrose transporter (OsSUT1-2) is required for heavy panicle rice to transfer and it’s frequency was 74% in *indica* rice^24^. Sugar transporters, SUT and SWEET groups for sucrose and monosaccharide sugar transporter (MST) group, transport glucose and fructose in the tissues of rice grain^25^. Cytoplasmic invertase converts sucrose into glucose and fructose which are activated (having one more high energy bonds) by the addition of phosphate by hexokinase or UDP by UDP glucose pyrophosphorylase or ADP by ADP glucose pyrophosphorylase (AGPase), fructose-6-phosphate isomerizes to glucose-6-phosphate by phosphor glucomutase and these activated monomers are converted to starch in amyloplast^26^. Amylose is synthesized by granule-bound starch synthases (GBSS) and amylopectin is synthesized by multiple enzymes—starch synthase (SS), starch-branching enzymes (SBEs) and starch-debranching enzymes (SDBEs) using the primer synthesized by starch phosphorylase^27^. SDBE or isoamylase provides crystallinity to the starch granule^28^. The availability of these starch synthesis genes in the genome or their expression during grain filling may vary among the rice varieties leading to variation in the structure of starch molecules. As the functions of these genes are known, following the expression of these genes during grain filling may explain the differential cooked grain quality among similar amylose containing rice varieties.

In source-sink studies, relation to DAF were examined for heat stress^29^ and drought stress^30^. The accumulation of amylose (iodine blue method) and amylopectin (by subtracting amylose from total starch) during grain filling were analysed in powders obtained by grinding multiple grains^30,31^. Amylose and amylopectin can be estimated by various methods^9^ and the estimated values also depend on the method employed for their estimation.

It is apparent from the above that amylose and amylopectin contents are yet to be estimated in individual grains. Similarly, the role of expression of starch metabolizing genes were yet to be studied in relation to grain cooking quality which is measured in terms of amylose content, gel consistency and alkali spreading value. Hence, considering the importance of individual grain weight during grain filling, the objective of this study includes the change in the quantities of amylose and amylopectin in individual grains in relation to the grain weight in varieties that differ in yield or grain quality at various stages of grain filling. In addition, expression of selective genes involved in starch synthesis was quantified and compared with amylose content, amylopectin content, gel consistency and alkali spreading value.

## Results

### Gas exchange parameters

ANOVA indicates that the variation in the gas exchange parameters was significant (P 0.001) among the genotypes as well as at various DAF’s of grain filling (Table 1). The photosynthetic rate (A), stomatal conductance (g_s_) and transpiration rate (E) values were highest at 5th DAF in all the four varieties used for yield (Table 2) and decreased in the subsequent DAF’s. At 5th DAF, A was significantly highest in Rasi followed by Ganjeekalli, HRC392 and Kalanamak. The photosynthetic rate in Rasi (one of the good yielders) was more than twice that of Kalanamak and 1.5 folds over HRC392. The other good yielder (Ganjeekalli) also noted photosynthetic rate nearly twice over kalanamak. However, the value was only five µmol/(m^2^ s) higher than HRC392 which also showed five µmol/(m^2^ s) higher value over Kalanamak. More notably, A decreased significantly to below detection level (BDL) by 20th DAF in poor yielders (HRC392 and Kalanamak). During the same period, A decreased nearly three folds in the good yielders (Rasi and Ganjeekalli). Although a negligible value, A was observed even at 30th DAF in Rasi while it reached to BDL by 25th DAF in Ganjeekalli.

**Table 1:** Two-way ANOVA of panicle and photosynthetic parameters in high and poor yielding rice. Significant at *P* 0.001 (***), *P* 0.01 (**) and *P* 0.05 (*).

Parameter		Sum Sq	Mean Sq	F value
Panicle length (cm)	Genotype	310.72	103.57	80.391***
	DAF	10.22	10.22	7.934*
	Residuals	24.48	1.29	
Total length of the branches (cm)	Genotype	33,882	11,294	6.359**
	DAF	2987	2987	1.682
	Residuals	33,745	1776	
Total number of branches	Genotype	12.394	4.131	5.936**
	DAF	2.935	2.935	4.217
	Residuals	13.222	0.696	
Total filled grain number	Genotype	57,501	19,167	13.22***
	DAF	23,069	23,069	15.91***
	Residuals	27,549	1450	
A [µmol/(m^2^ s)]	Genotype	226.2	75.4	7.975***
	DAF	2641.5	2641.5	279.357***
	Residuals	595.7	9.5	
gS [mol/(m^2^ s)]	Genotype	0.3535	0.1178	8.337***
	DAF	0.9809	0.9809	69.399***
	Residuals	0.8905	0.0141	
Ci [µmol/mol]	Genotype	213,060	71,020	11.5***
	DAF	40,861	40,861	6.617*
	Residuals	389,058	6176	
E [mmol/(m^2^ s)]	Genotype	109.6	36.5	16.11***
	DAF	318.8	318.8	140.54***
	Residuals	142.9	2.3

**Table 2:** Variation in panicle and photosynthetic parameters in high and poor yielding rice.

Variety	DAF	Panicle length (cm)	Total length of branches (cm)	Total number of branches	Total filled grain number	A [µmol/ (m^2^ s)]	g_S_ [mol/ (m^2^ s)]	Ci [µmol/ mol]	E [mmol/ (m^2^ s)]
Ganjeekalli	5	21.8 ± 1.1^cde^	98.0 ± 1.8^abcde^	9.5 ± 0.7^abcd^	12.0 ± 1.6^hi^	21.3 ± 1.5^b^	0.7 ± 0.2^a^	310.9 ± 11.7^b^	9.2 ± 0.5^b^
	10	21.5 ± 0.7^cde^	82.5 ± 1.2^cdefgh^	9.0 ± 0.1^abcde^	78.5 ± 2.2^efg^	8.0 ± 7.0^gh^	0.1 ± 0.1^fghi^	249.7 ± 46.6^de^	4.2 ± 2.5f.
	15	21.3 ± 1.0^cde^	81.4 ± 1.6^cdefgh^	8.5 ± 0.7^bcdef^	88.0 ± 3.5^efg^	10.7 ± 1.2^efg^	0.2 ± 0.0^def^	250.2 ± 11.5^de^	6.4 ± 0.3^de^
	20	22.0 ± 2.8^ cd^	98.1 ± 1.3^abcde^	9.0 ± 0.1^abcde^	117.5 ± 3.0^cde^	6.8 ± 0.4^hi^	0.2 ± 0.1^def^	296.9 ± 23.6^bc^	4.7 ± 1.4^ef^
	25	18.0 ± 1.4^ fg^	64.0 ± 2.^ghij^	7.0 ± 1.4^ef^	88.5 ± 5.6^efg^	BDL	BDL	BDL	BDL
	30	20.4 ± 0.4^def^	77.7 ± 1.4^defgh^	8.0 ± 0.1^cdef^	108.0 ± 3.8^de^	BDL	BDL	BDL	BDL
HRC392	5	22.6 ± 1.2^ cd^	79.7 ± 0.9^cdefgh^	9.5 ± 0.7^abcd^	6.0 ± 0.8^i^	17.0 ± 0.6^c^	0.2 ± 0.0^ cd^	240.7 ± 13.4^de^	8.0 ± 0.4^bc^
	10	22.3 ± 1.1^ cd^	75.9 ± 1.2^defghi^	9.5 ± 0.7^abcd^	84.5 ± 2.6^efg^	15.2 ± 0.6^ cd^	0.3 ± 0.0^c^	267.7 ± 7.7^ cd^	7.9 ± 0.7^bc^
	15	20.6 ± 1.3^def^	66.7 ± 1.2^fghij^	8.5 ± 0.7^bcdef^	83.0 ± 1.7^efg^	2.8 ± 1.2^kj^	0.1 ± 0.0^ghij^	310.6 ± 11.8^b^	2.6 ± 0.8^gh^
	20	21.0 ± 1.4^cdef^	65.0 ± 0.8^fghij^	9.0 ± 1.4^abcde^	80.5 ± 2.7^efg^	BDL	BDL	BDL	BDL
	25	20.6 ± 0.7^cdef^	73.8 ± 1.1^efghi^	10.0 ± 0.1^abc^	102.0 ± 3.3^def^	BDL	BDL	BDL	BDL
	30	21.7 ± 1.2^cde^	90.7 ± 1.9^bcdef^	10.3 ± 2.3^ab^	150.7 ± 4.8^bcd^	BDL	BDL	BDL	BDL
Kalanamak	5	27.4 ± 1.3^a^	116.3 ± 1.6^ab^	11.0 ± 0.1^a^	35.5 ± 4.1^ghi^	12.2 ± 0.7^de^	0.1 ± 0.0^efghi^	201.9 ± 23.0^ fg^	4.3 ± 0.5f.
	10	26.4 ± 2.7^ab^	106.8 ± 2.0^abc^	9.0 ± 0.1^abcde^	176.5 ± 10.4^bc^	11.3 ± 1.0^def^	0.1 ± 0.0^efgh^	220.8 ± 28.2^ef^	4.6 ± 0.8^ef^
	15	25.9 ± 3.7^ab^	120.4 ± 1.2^a^	10.0 ± 2.8^abc^	245.5 ± 6.3^a^	3.2 ± 1.0^jk^	0.05 ± 0.0^ij^	182.6 ± 55.3^ g^	1.5 ± 0.5^ h^
	20	26.0 ± 2.8^ab^	98.2 ± 2.4^abcde^	9.5 ± 2.1^abcd^	209.0 ± 9.6^ab^	BDL	BDL	BDL	BDL
	25	23.8 ± 0.4^bc^	83.1 ± 1.2^cdefg^	8.0 ± 0.0^cdef^	160.5 ± 5.9^bcd^	BDL	BDL	BDL	BDL
	30	26.2 ± 1.4^ab^	101.3 ± 1.1^abcd^	8.3 ± 1.2^cdef^	246.3 ± 8.6^a^	BDL	BDL	BDL	BDL
Rasi	5	15.8 ± 1.8^gh^	67.2 ± 1.2^fghij^	9.0 ± 1.4^abcde^	11.0 ± 2.1^hi^	26.5 ± 0.5^a^	0.7 ± 0.1^a^	320 ± 14.2^b^	11.2 ± 0.4^a^
	10	18.8 ± 0.4^ef^	62.3 ± 1.0^ghij^	7.0 ± 0.1^ef^	57.5 ± 1.8^efghi^	22.9 ± 0.8^b^	0.6 ± 0.1^b^	294.1 ± 16.9^bc^	8.0 ± 0.3^bc^
	15	15.5 ± 0.1^gh^	53.8 ± 0.7^hij^	8.0 ± 1.4^cdef^	53.5 ± 1.6^efghi^	13.9 ± 1.2^d^	0.2 ± 0.0^cde^	241.9 ± 12.8^de^	7.5 ± 0.7^ cd^
	20	14.3 ± 1.1^ h^	42.5 ± 1.0^j^	6.5 ± 0.7f.	39.0 ± 1.5^fghi^	9.4 ± 1.6^fgh^	0.1 ± 0.0^defg^	249.5 ± 27.6^de^	5.9 ± 0.8^de^
	25	14.6 ± 1.9^ h^	48.5 ± 0.9^ij^	7.5 ± 0.7^def^	51.5 ± 2.0^efghi^	4.7 ± 1.0^ij^	0.1 ± 0.0^fghi^	312.0 ± 17.4^b^	3.5 ± 0.4^ fg^
	30	15.8 ± 1.0^gh^	57.7 ± 0.9^ghij^	8.0 ± 0.1^cdef^	69.3 ± 2.1^efgh^	0.6 ± 0.2^ki^	0.05 ± 0.0^hij^	347.5 ± 12.8^a^	1.8 ± 1.0^ h^
LSD @ 0.05		1.3	11.57	0.88	33.07	2.75	0.08	33.45	1.25

A: photosynthetic rate, g_S :_ stomatal conductance, Ci : intercellular CO_2_, E: transpiration rate, BDL: below detection level.

Different letters after each data point indicate statistically significant differences at *p* < 0.05. Data point with no common letters are significantly different (*p* < 0.05).

The gS values were also highest in both Rasi and Ganjeekalli. The E was highest in Rasi followed by Ganjeekalli, HRC392 and Kalanamak. The E decreased to BDL by 20th DAF in HRC392 and Kalanamak and during the same period, E decreased by two folds in both Rasi and Ganjeekalli. Green colour was observed in the flag leaves of Rasi even at 30th DAF.

### Panicle topology and grain weight

Among the four varieties used for yield variation, ANOVA indicates that the variation in panicle length, total length of the branches, total number of branches and total filled grain number were significant (*P* 0.001) among the genotypes (Table 1). In the case of DAF’s, variation in panicle length and total filled grain number were significant at 0.05 and 0.001 probability respectively. Panicle length varied significantly from 14.3 cm at 20th DAF in Rasi to 27.4 cm at 5th DAF in Kalanamak (Table 2). The total number of branches also varied significantly varied from 6.5 at 20th DAF in Rasi to 11 at 5th DAF in Kalanamak. The total length of branches within the panicle also varied significantly from 42.5 at 20th DAF in Rasi to 116.3 cm at 5th DAF in Kalanamak. The total number of filled grains in the panicle also varied significantly from 11.0 at 5th DAF in Rasi to 246.3 at 30th DAF in Kalanamak.

**Figure 1:**
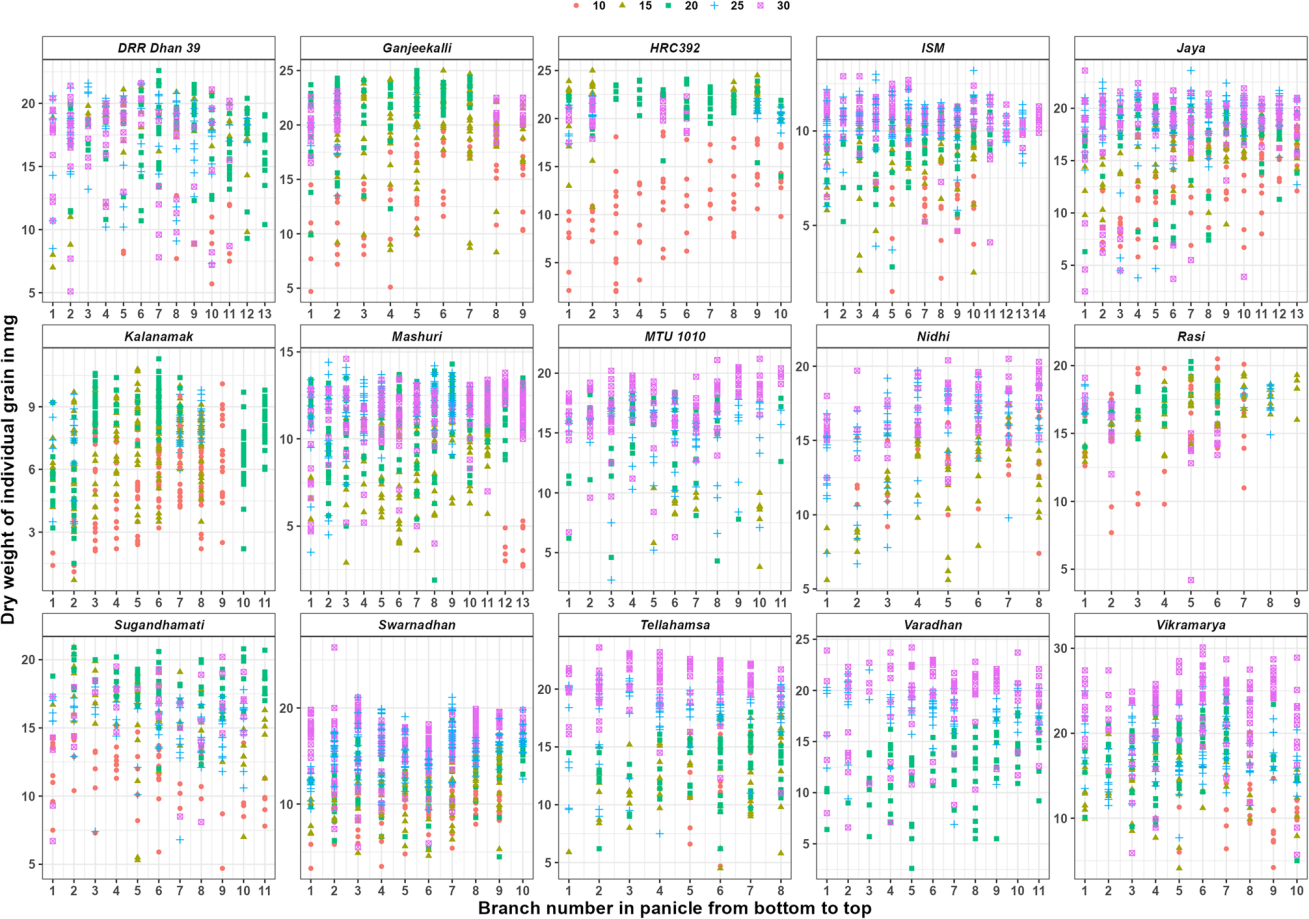
Dry weight of individual grains in various branches of the panicle of the varieties collected at various DAF’s. Legend represents the colour used for each DAF.

At various DAF’s within a genotype, panicle length varied from 14.3 to 15.8 cm in Rasi, 18 to 21.8 cm in Ganjeekalli, 20.6 to 22.6 cm in HRC392 and 23.8 to 27.4 cm in Kalanamak. The total number of branches varied from 6.5 to 9.0 in Rasi, 7 to 9.5 in Ganjeekalli, 8.5 to 10.3 in HRC392 and 8.0 to 11.0 in Kalanamak. The total number of branches within the panicle significantly varied from 42.5 to 67.2 cm in Rasi, 64.0 to 98.1 cm in Ganjeekalli, 65.0 to 90.7 cm in HRC392 and 83.1 to 116.3 in Kalanamak. The total number of filled grains in the panicle varied from 11.0 to 69.3 in Rasi, 12 to 117.5 in Ganjeekalli, 6.0 to 150.7 in HRC392 and 35.5 to 246.3 in Kalanamak (Table 2, Fig. 1). Therefore, the panicle of Kalanamak noted highest number of branches, number of grains and total branch length which inturn resulted in highest (2.43) grain density (ratio of total grain to total length of the branches) in this genotype followed by HRC392 (1.66), Ganjeekalli (1.39) and Rasi (1.20).

ANOVA indicates that the variation in grain dry weight of the individual grains was significant (P 0.001) among the genotypes (used for yield as well as for grain quality), DAF’s and various branches of the panicle (Table 3). Among the four varieties studied for yield, grain dry weight was least in Kalanamak (Table 4) and it was similar in the remaining three varieties (Rasi, Ganjeekalli and HRC392). Further, the cumulative grain dry weight at 30th DAF was highest for Ganjeekalli which is good in grain yield. At different DAF’s, in Ganjeekalli, the individual grain weight significantly varied from 4.7 at 10th DAF to 24.3 mg at 20th DAF and the mean individual grain weight was least (13.7 mg) at 10th DAF, increased to 18.4 mg and reached to maximum (around 20.0 mg) by 20th DAF. In Rasi, the individual grain weight significantly varied from 4.2 at 30th DAF to approximately 20.0 mg at all DAF’s and the mean individual grain weight was least (14.8 mg) at 30th DAF and reached to maximum (16 to 17.0 mg) at other DAF’s. In HRC392, the individual grain weight significantly varied from 2.1 at 10th DAF to 25.0 mg at 15th DAF and the mean individual grain weight was least (11.7 mg) at 10th DAF and reached to maximum (around 20.0 mg) by 15th DAF. In Kalanamak, the individual grain weight significantly varied from 0.7 at 15th DAF to maximum (around 10.0 mg) at other DAF’s and the mean individual grain weight was least (5.7 mg) at 10th DAF and reached to maximum (around 7.0 mg) at other DAF’s.

**Table 3:** Two-way ANOVA of individual grain dry weight and starch components during grain filling at various DAF’s. Significant at *P* 0.001 (***) and *P* 0.01 (**).

Parameter		Sum Sq	Mean Sq	F value
Grain weight (mg)	Genotype	77,198	5514	550.9***
	DAF’s	16,679	16,679	1666.3***
	Branch	1304	1304	130.3***
	Residuals	61,199	10	
Amylose content (mg) in single grain	Genotype	709.5	47.3	150.63***
	DAF’s	76.2	76.2	242.53***
	Branch	41.4	3.2	10.14***
	Individual grain weight	687.3	687.3	2188.87***
	Residuals	612.6	0.3	
Amylopectin content (mg) in single grain	Genotype	6752	450	216.99***
	DAF’s	840	840	405.12***
	Branch	316	24	11.72***
	Individual grain weight	4109	4109	1980.93***
	Residuals	4047	2	
Amylose content (%) in single grain	Genotype	4117	274.5	16.698***
	DAF’s	25	24.9	1.514
	Branch	539	41.5	2.523**
	Individual grain weight	1345	1345.2	81.839***
	Residuals	32,069	16.4	
Amylopectin content (%) in single grain	Genotype	81,340	5423	47.044***
	DAF’s	3693	3693	32.041***
	Branch	8483	653	5.661***
	Individual grain weight	8768	8768	76.064***
	Residuals	224,887	115

**Table 4:** Grain weight, amylose and amylopectin contents in single grain at various intervals grain filling.

Name	Grain weight (mg) DAF							Amylose content (mg) in single grain						Amylopectin content (mg) in single grain					
		10th	15th	20th	25th	30th	LSD @ 0.05	10th	15th	20th	25th	30th	LSD @ 0.05	10th	15th	20th	25th	30th	LSD @ 0.05
DRR Dhan 39	Mean	9.3^b^	17.8^a^	17.2^a^	17.5^a^	16.5^a^	12.62	1.01^a^	1.82^b^	2.22^a^	2.37^a^	–	2.98	1.16^c^	2.48^c^	4.25^b^	6.59^a^	–	5.92
	Min	5.7	7	9.3	7.3	5.1		0.60	0.76	0.06	0.64	–		0.56	1.14	0.44	2.86	–	
	Max	12.7	21.7	22.6	21.6	21.6		1.38	2.97	4.47	4.03	–		1.73	3.91	8.53	11.27	–	
Ganjeekalli	Mean	13.7^c^	18.4^b^	20.3^a^	20.1^a^	20.5^a^	12.13	1.9^b^	3.4^a^	3.6^a^	3.5^a^	1.8^b^	4.03	7.1^c^	7.0^c^	8.3^b^	8.7^b^	10.0^a^	6.87
	Min	4.7	8.3	9.9	13.5	16.5		0.3	1.2	1.1	2.3	0.8		2.7	3.3	4.2	6.4	3.2	
	Max	19.9	22.2	24.3	22.9	23.1		3.3	4.6	4.7	4.1	4.4		13.6	9.3	10.8	11.0	14.1	
HRC392	Mean	11.7^c^	21.7^a^	20.9^ab^	20.5^b^	20.4^b^	13.01	1.8^c^	3.5^b^	3.9^a^	3.7^ab^	1.9^c^	3.96	5.9^b^	8.6^a^	8.4^a^	8.6^a^	8.4^a^	7.83
	Min	2.1	10.8	14.0	17.4	17.5		0.2	1.3	2.5	3.1	1.3		1.0	2.1	5.8	5.2	3.9	
	Max	17.9	25.0	23.9	22.4	22.5		3.4	5.0	4.8	4.6	3.1		12.1	11.8	11.6	11.0	12.6	
ISM	Mean	6.8^c^	8.4^b^	9.1^ab^	10.3^a^	10.6^a^	4.53	0.86^bc^	0.98^b^	1.04^b^	1.35^a^	1.22^a^	0.65	2.34^c^	2.58^c^	3.23^b^	3.33^b^	3.70^a^	2.57
	Min	1.5	2.5	2.8	3.7	4.1		0.20	0.27	0.34	0.32	0.41		0.46	0.59	0.73	0.68	1.08	
	Max	11	11.3	11.8	13.2	12.9		1.67	1.56	1.88	1.95	2.07		4.35	4.66	5.54	4.50	5.58	
Jaya	Mean	13.8^c^	16.3^bc^	17^b^	18.8^a^	17.8^ab^	15.57	1.05^b^	1.30^b^	1.08^b^	2.06^a^	2.04^a^	2.64	8.57^b^	10.73^a^	10.21^a^	9.70^ab^	8.30^b^	10.68
	Min	5.8	7.4	6.3	3.8	2.5		0.06	0.17	0.02	0.25	0.17		4.46	6.62	4.85	2.20	1.43	
	Max	18.9	20.3	21.3	23.6	23.6		2.32	2.64	2.22	3.16	4.94		15.13	15.34	15.95	14.29	13.66	
Kalanamak	Mean	5.7^c^	7.2^ab^	6.7^b^	7.7^a^	–	5.65	0.8^b^	0.8^b^	1.2^a^	1.1^a^	–	1.16	2.8^bc^	3.0^b^	3.7^a^	2.7^c^	–	3.52
	Min	1.1	0.7	1.5	3.4	–		0.1	0.1	0.2	0.3	–		0.6	0.4	0.6	0.8	–	
	Max	10.1	10.0	9.8	9.8	–		3.8	2.0	1.6	1.6	–		7.0	6.7	6.1	4.1	–	
Mashuri	Mean	3.9^c^	9^b^	11^a^	11.8^a^	11.5^a^	9.7	0.38^b^	1.23^a^	1.35^a^	1.35^a^	0.97^b^	1.7	1.24^d^	3.15^c^	4.10^bc^	5.95^a^	5.01^ab^	5.7
	Min	2.7	2.9	1.9	3.5	4		0.25	0.02	0.10	0.28	0.31		0.72	0.05	0.92	1.92	2.07	
	Max	5.3	12.9	14.3	14.4	14.6		0.51	2.04	2.25	2.35	1.55		1.54	5.77	8.19	8.61	7.39	
MTU 1010	Mean	–	8.7^c^	15.2^b^	14.7^bc^	17.1^a^	13.31	–	0.93^b^	1.85^a^	1.75^a^	1.64^a^	2.29	–	3.79^c^	6.29^b^	5.65^b^	7.51^a^	6.85
	Min	–	3.8	4.3	2.7	12.2		–	0.48	0.50	0.18	0.41		–	1.30	1.82	1.53	3.00	
	Max	–	10.4	18.5	18.4	20.5		–	1.62	2.97	2.76	3.01		–	5.32	9.57	9.09	11.15	
Nidhi	Mean	14.2^b^	12.3^c^	–	15.7^b^	17.1^a^	11.03	1.88^a^	1.27^b^	–	1.80^a^	2.09^a^	1.8	7.85^a^	6.35^b^	–	6.55^b^	6.66^b^	5.3
	Min	7.4	5.6	–	6.7	12.2		0.88	0.37	–	1.11	1.29		4.62	2.75	–	4.12	5.13	
	Max	18.3	17.5	–	19.7	20.5		2.61	2.27	–	2.85	3.21		10.30	12.20	–	12.44	7.58	
Rasi	Mean	15.9^b^	16.5^ab^	17.3^a^	17.2^a^	14.8^c^	11.97	3.0^a^	2.5^a^	2.6^a^	2.3^ab^	2.7^a^	3.25	7.1^b^	6.3^c^	6.8^bc^	7.6^b^	9.3^a^	7.48
	Min	7.7	12.9	14.0	14.9	4.2		1.3	1.9	2.0	1.2	0.8		3.7	4.2	5.2	5.7	5.0	
	Max	20.5	19.3	20.3	18.6	19.1		4.3	3.1	3.4	3.5	3.4		10.5	8.1	8.2	9.2	12.2	
Sampada	Mean	7.6^c^	12.2^b^	14^a^	11.3^bc^	12.2^b^	9.75	1.0^b^	1.4^a^	–	1.2^ab^	1.1^ab^	1.46	3.6^b^	6.0^a^	–	4.0^b^	5.6^a^	5.48
	Min	1.7	3.6	8.3	3	5.7		0.4	0.3	–	0.4	0.5		1.0	2.1	–	1.9	3.4	
	Max	14.3	14.5	16.2	15.6	15.1		1.9	2.1	–	2.1	1.8		5.5	7.9	–	5.6	8.8	
Sungandhamati	Mean	11.5^c^	15.8^b^	18^a^	15^b^	15.9^b^	10.49	1.05^c^	2.42^a^	2.09^b^	2.46^a^	–	2.11	4.95^b^	7.67^a^	7.40^a^	5.13^b^	–	5.37
	Min	4.7	5.3	12	6.8	6.7		0.36	0.41	0.62	1.01	–		1.73	3.00	3.19	1.64	–	
	Max	17.9	20.9	20.9	18	20.3		2.09	3.55	3.28	3.53	–		7.31	10.10	11.15	7.21	–	
Swarnadhan	Mean	9.8^b^	10.8^b^	13.6^a^	15.5^a^	16.3^a^	11.59	1.12^b^	2.10^a^	1.20^b^	1.97^a^	2.08^a^	2.36	4.20^c^	4.99^b^	5.59^ab^	6.5^a^	5.79^ab^	5.81
	Min	3.3	4.6	4.5	9.4	5.5		0.32	0.34	0.43	0.36	1.16		1.52	0.40	2.59	2.28	3.21	
	Max	15.4	16.7	18.7	21.1	26.3		2.30	3.34	2.15	2.92	2.89		6.80	8.05	9.38	10.30	8.65	
Tellahamsa	Mean	12.1^c^	12.7^c^	13.2^c^	18.7^b^	20.3^a^	12.17	1.14^c^	1.17^c^	1.33^c^	2.19^b^	2.74^a^	2.18	5.85	5.68	6.53	6.89	7.19	5.86
	Min	4.7	4.5	6.2	7.5	11		0.29	0.51	0.52	0.58	1.56		1.77	2.42	3.64	2.84	4.93	
	Max	16.9	17.8	18.3	23.6	22.6		2.11	1.90	2.56	4.36	3.63		8.62	9.37	9.82	15.35	9.48	
Varadhan	Mean	–	–	12^b^	17.8^a^	18.7^a^	13.63	–	–	1.50^b^	1.91^a^	1.50^b^	1.98	–	–	5.23^b^	6.26^a^	6.43^a^	5.58
	Min	–	–	2.6	6.9	6.6		–	–	0.57	0.45	0.39		–	–	2.90	2.24	2.51	
	Max	–	–	18.4	22	24.2		–	–	2.57	2.76	2.28		–	–	8.44	7.99	9.65	
Vikramarya	Mean	10.6^d^	14.9^c^	18.3^bc^	17.6^ab^	22.7^a^	17.02	1.08^d^	1.78^c^	2.59^b^	2.55^b^	3.22^a^	3.06	5.04^c^	7.42^b^	6.64^bc^	7.63^b^	9.29^a^	8.95
	Min	4.2	4.1	5	7.7	5.7		0.07	0.42	0.40	0.59	0.67		0.69	1.43	1.92	2.54	2.32	
	Max	15.1	21.8	24.4	26.6	30.1		1.62	2.97	4.22	4.04	5.00		7.60	10.84	9.67	11.86	13.99	

Different letters after each data point indicate statistically significant differences at *p* < 0.05. Data point with no common letters are significantly different (*p* < 0.05).

Apart from the above, the individual grain weight was also observed among the other varieties that tested for grain quality (Table 4). At different DAF’s, in DRR Dhan 39, the individual grain weight significantly varied from 5.1 at 30th DAF to 22.6 mg at 20th DAF and the mean individual grain weight was least (9.3 mg) at 10th DAF and reached to maximum (around 17.0 mg) at other DAF’s. In ISM, it significantly varied from 1.5 at 10th DAF to 13.2 mg at 25th DAF and it’s mean was least (6.8 mg) at 10th DAF, increased to 8.4 mg at 15th DAF followed by 9.1 mg at 20th DAF and reached to maximum (10.0 mg) by 25th DAF. In Jaya, it significantly varied from 2.5 at 30th DAF to 23.6 mg by 25th DAF and it’s was least (13.8 mg) at 10th DAF, increased to 16.3 mg and reached to maximum (18.8 mg) by 25th DAF. In Mashuri, it significantly varied from 1.9 at 20th DAF to 14.6 mg at 30th DAF and it’s was least (3.9 mg) at 10th DAF, increased to 9.0 mg at 15th DAF and reached to maximum (around 11.0 mg) by 20th DAF. In MTU 1010, it significantly varied from 2.7 at 25th DAF to 20.5 mg at 30th DAF and it's mean was least (8.7 mg) at 15th DAF, increased to around 15.0 mg at 20th DAF and reached to maximum (17.1 mg) by 30th DAF. In Nidhi, it significantly varied from 5.6 at 15th DAF to around 20.0 mg by 25th DAF and its mean was least (12.3 mg) at 15th DAF, increased to around 15.0 mg and reached to maximum (17.1 mg) by 30th DAF. In Sampada, it significantly varied from 1.7 at 10th DAF to around 16.0 mg by 20th DAF and its mean was least (7.6 mg) at 10th DAF, increased to around 12.0 mg and reached to maximum (14.0 mg) by 20th DAF. In Sugandhamati, it significantly varied from 4.7 at 10th DAF to 20.9 mg by 15th DAF and its mean was least (11.5 mg) at 10th DAF, increased to 15.8 mg by 15th DAF and reached to maximum (18.0 mg) by 20th DAF. In Swarnadhan, it significantly varied from 3.3 at 10th DAF to 26.3 mg at 30th DAF and its mean was least (9.8 mg) at 10th DAF and reached to maximum (around 16.0 mg) by 20th DAF. In Tellahamsa, it significantly varied from 4.7 at 10th DAF to 24.3 mg at 20th DAF and its mean was least (13.7 mg) at 10th DAF, increased to 18.4 mg and reached to maximum (around 20.0 mg) by 20th DAF. In Varadhan, it significantly varied from 2.6 at 20th DAF to 24.2 mg at 30th DAF and it’s was least (12.0 mg) at 10th DAF and reached to maximum (around 18.0 mg) by 25th DAF. In Vikramarya, it significantly varied from 4.1 at 15th DAF to 30.1 mg at 30th DAF and its mean was least (10.6 mg) at 10th DAF, increased to 14.9 mg by 15th DAF and reached to maximum (around 18.0 mg) by 20th DAF.

### Relation between starch components and grain weight

ANOVA indicates that the variation in amylose content in single grain, amylopectin content in single grain, amylose content in percentage in single grain and amylopectin content in percentage in single grain were significant (P 0.001) among the genotypes, DAF’s, branches and individual grain dry weight (Table 3).

During grain filling, among the four varieties studied for yield, the mean amylose content in individual grain in the two poor yielders- Kalanamak and HRC392 significantly varied from 0.8 to 1.2 mg and 1.8 to 3.9 mg respectively (Table 2). While in the two good yielders- Ganjeekalli and Rasi, it significantly varied from 1.8 to 3.6 mg and 2.3 to 3.0 mg respectively. The mean amylopectin content in individual grain in Kalanamak and HRC392 ranged from 2.7 to 3.7 mg and 5.9 to 8.6 mg respectively. While in Ganjeekalli and Rasi it ranged from 7.1 to 10.0 mg and 6.3 to 9.3 mg respectively. The accumulation of amylose and amylopectin quantities were more during the first 15 days of the grain filling period and marginal increments were observed in the subsequent intervals (Table 3).

Among the other varieties that tested for grain quality, the mean amylose content in individual grain significantly varied from 1.01 (at 10th DAF) to 2.37 mg (at 25th DAF) in DRR Dhan 39, 0.86 ( at 10th DAF) to 1.35 mg (at 25th DAF) in ISM, 1.05 (10th DAF) to 2.06 mg (at 25th DAF) in Jaya, 0.38 (at 10th DAF) to 1.35 mg (at 20th DAF) in Mashuri, 0.93 (at 15th DAF) to 1.85 mg (at 20th DAF) in MTU 1010, 1.27 (at 15th DAF) to 2.09 mg (at 30th DAF) in Nidhi, 2.3 (at 25th DAF) to 3.0 mg (at other DAF’s) in Rasi, 1.0 (at 10th DAF) to 1.4 mg (at 15th DAF) in Sampada, 1.05 (at 10th DAF) to 2.46 mg (at 25th DAF) in Sugandhamati, 1.12 (at 10th and 20th DAF’s) to around 2.0 mg (in other DAF’s) in Swarnadhan, 1.14 (at 10th, 15th and 20th DAF’s) to 2.74 mg (at 30th DAF) in Tellahamsa, 1.5 (at 20th and 30th DAF’s) to 1.9 mg (at 25th DAF) in Varadhan and 1.08 (at 10th DAF) to 3.22 mg (at 30th DAF) in Vikramarya.

While the range of amylose content in individual grain significantly varied from 0.06 (at 20th DAF) to 4.47 mg (at 20th DAF) in DRR Dhan 39, 0.2 (10th DAF) to 2.07 mg (at 30th DAF) in ISM, 0.06 (10th DAF) to 4.94 mg (at 30th DAF) in Jaya, 0.02 (at 15th DAF) to 2.35 mg (at 25th DAF) in Mashuri, 0.18 (at 25th DAF) to 3.01 mg (at 30th DAF) in MTU 1010, 0.37 (at 15th DAF) to 3.21 mg (at 30th DAF) in Nidhi, 0.8 (at 30th DAF) to 4.3 mg (at 10 DAF) in Rasi, 0.3 (at 15th DAF) to 2.1 mg (at 15th and 25th DAF’s) in Sampada, 0.36 (at 10th DAF) to 3.55 mg (at 15th DAF) in Sugandhamati, 0.32 (at 10th DAF) to 3.34 mg (at 15th DAF) in Swarnadhan, 0.29 (at 10th DAF) to 4.36 mg (at 25th DAF) in Tellahamsa, 0.39 (at 30th DAF) to 2.76 mg (at 25th DAF) in Varadhan and 0.07 (at 10th DAF) to 5.00 mg (at 30th DAF) in Vikramarya.

The mean amylopectin content in individual grain significantly varied from 1.16 (at 10th DAF) to 6.59 mg (at 25th DAF) in DRR Dhan 39, 2.34 (at 10th DAF) to 3.7 mg (at 30th DAF) in ISM, around 8.5 (10th and 30th DAF’s) to around 10.5 mg (at 15th and 20th DAF’s) in Jaya, 1.24 (at 10th DAF) to 5.95 mg (at 25th DAF) in Mashuri, 3.79 (at 15th DAF) to 7.51 mg (at 30th DAF) in MTU 1010, 6.35 (at 15th DAF) to 7.85 mg (at 10th DAF) in Nidhi, 6.3 (at 15th DAF) to 9.3 mg (at 30th DAF) in Rasi, 3.6 (at 10th DAF) to 6.0 mg (at 15th and 30th DAF’s) in Sampada, 4.95 (at 10th DAF) to 7.67 mg (at 15th and 20th DAF’s) in Sugandhamati, 4.2 (at 10th DAF) to 6.5 mg (at 25th DAF) in Swarnadhan, 5.68 (at 15th DAF) to 7.19 mg (at 30th DAF) in Tellahamsa, 5.23 (at 20th DAF) to 6.43 mg (at 30th DAF) in Varadhan and 5.04 (at 10th DAF) to 9.29 mg (at 30th DAF) in Vikramarya.

While the range of amylopectin content in individual grain significantly varied from 0.44 (at 20th DAF) to 11.27 mg (25th DAF) in DRR Dhan 39, 0.46 (10th DAF) to 5.58 mg (at 30th DAF) in ISM, 1.43 (30th DAF) to 15.95 mg (at 20th DAF) in Jaya, 0.05 (at 15th DAF) to 8.61 mg (at 25th DAF) in Mashuri, 1.3 (at 15th DAF) to 11.15 mg (at 30th DAF) in MTU 1010, 2.75 (at 15th DAF) to 12.44 mg (at 25th DAF) in Nidhi, 3.7 (at 15th DAF) to 12.2 mg (at 30th DAF) in Rasi, 1.0 (at 10th DAF) to 8.8 mg (at 30th DAF) in Sampada, 1.64 (at 25th DAF) to 11.15 mg (at 20th DAF) in Sugandhamati, 0.40 (at 15th DAF) to 10.3 mg (at 25th DAF) in Swarnadhan, 1.77 (at 10th DAF) to 15.35 mg (at 25th DAF) in Tellahamsa, 2.24 (at 20th DAF) to 9.65 mg (at 30th DAF) in Varadhan and 0.69 (at 10th DAF) to 13.99 mg (at 30th DAF) in Vikramarya.

**Figure 2:**
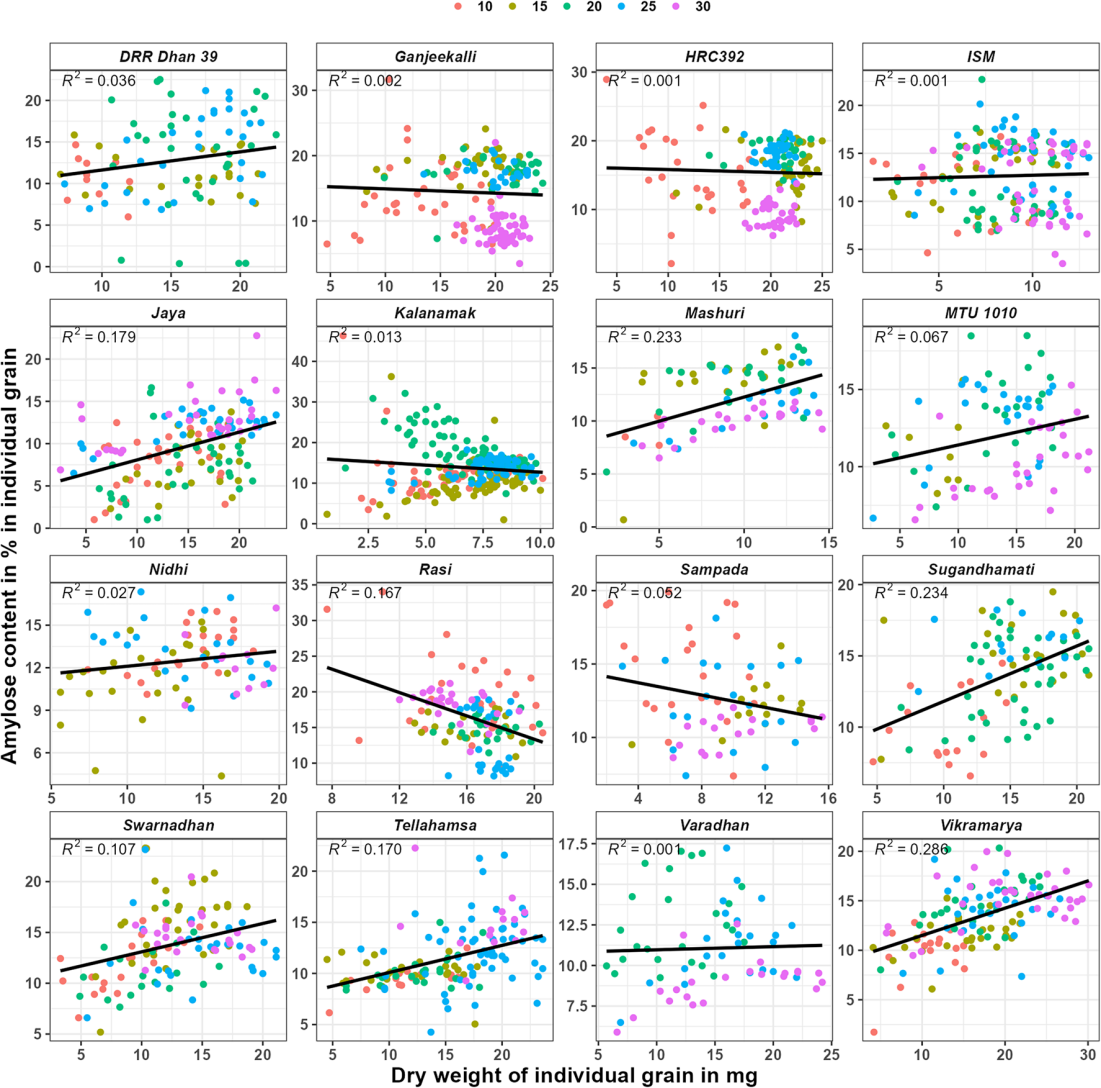
Amylose content in % in individual grains of rice varieties during grain filling. Legend represents the colour used for each DAF.

**Figure 3:**
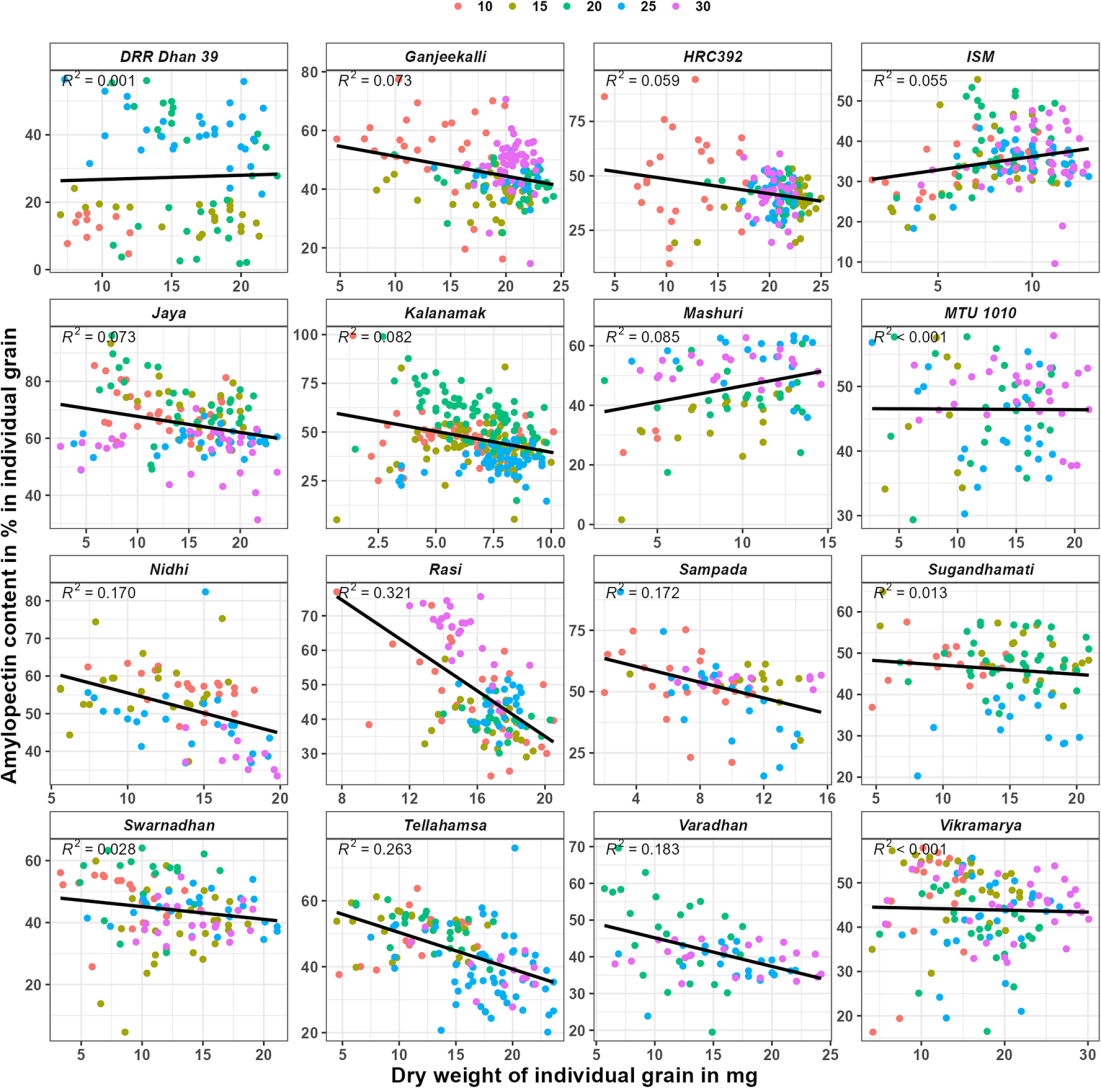
Amylopectin content in % in individual grains in rice varieties during grain filling. Legend represents the colour used for each DAF.

In amylose content in mg in individual grain vs dry weight in mg in individual grain graph (supplementary Fig. 1), the regression value (R^2^) was highly significant (> 0.7) in seven varieties, significant (> 0.5 and < 0.7) in four varieties and non-significant (< 0.5) in the remaining five varieties. Whereas in amylopectin content in mg in individual grain vs dry weight in mg in individual grain graph (supplementary Fig. 2), the R^2^ value was highly significant in eight varieties, significant in four varieties and non-significant in the remaining four varieties. These results indicate that the quantities of amylose and amylopectin increase with the increase in individual grain dry weight in all the varieties. However, no significant correlation was found in amylose content in% (Fig. 2) vs grain weight in mg in individual grain as well as amylopectin in% vs grain weight in mg in individual grain (Fig. 3) graphs.

In amylose content in mg in individual grain vs grain weight in mg in individual grain graph (Supplementary Fig. 3), the R^2^ value was either significant or highly significant in eleven, twelve, eleven, fifteen and eight varieties at 10th, 15th, 20th, 25th and 30th DAF’s respectively. Similarly, in amylopectin content in mg in individual grain vs grain weight in mg in individual grain graph (Supplementary Fig. 4), the R^2^ value was either significant or highly significant in eleven, thirteen, ten, twelve and seven varieties at 10th, 15th, 20th, 25th and 30th DAF’s respectively.

**Figure 4:**
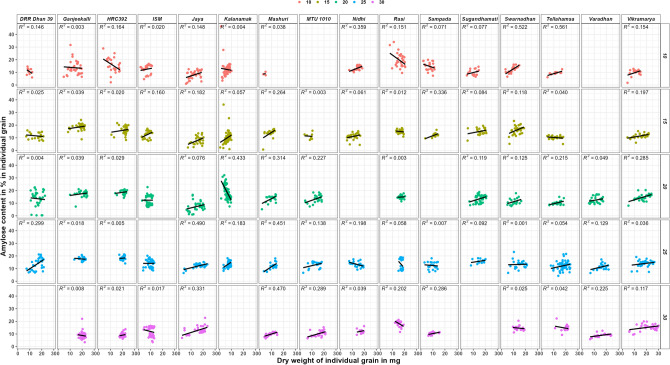
Amylose content in % in individual grains of rice varieties at various DAF’S of grain filling. Legend represents the colour used for each DAF.

In amylose content in % in individual grain vs grain weight in mg in individual grain (Fig. 4), the R^2^ value was non-significant for two (Ganjeekalli and Kalanamak) and significant (> 0.5) for Swarnadhan and Tellahamsa varieties at 10th DAF. It was non-significant (< 0.5) for all the varieties at other DAF’s. However, R line showed negative trend in four (DRR Dhan 39, HRC392, Rasi and Sampada) and positive trend in the remaining varieties at 10th DAF, negative trend in three (DRR Dhan 39, Rasi and Tellahamsa) and positive trend in the remaining varieties at 15th DAF, negative trend in Kalanamak and positive trend in the remaining varieties at 20th DAF, negative trend in three (Ganjeekalli, Nidhi and Rasi) and positive trend in the remaining varieties at 25th DAF and negative trend in four (ISM, Rasi, Swarnadhan and Tellahamsa) and positive trend in the remaining varieties at 30th DAF.

**Figure 5:**
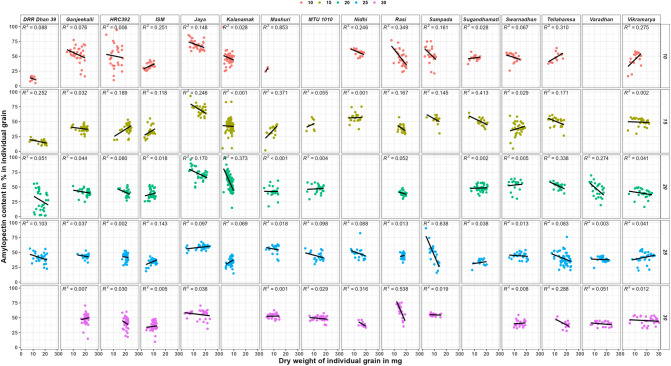
Amylopectin content in % in individual grains in rice varieties at various DAF’S of grain filling. Legend represents the colour used for each DAF.

In the case of amylopectin content in % in individual grain vs grain weight in mg in individual grain graph (Fig. 5), the R^2^ value was significant for Mashuri and non-significant for all the other varieties at 10th DAF, non-significant (< 0.5) for all the varieties at 15th, 20th and 25th DAF’s and significant for Rasi alone at 30th DAF. However, the R line showed positive trend in only five (ISM, Mashuri, Sugandhamati, Tellahamsa and Vikramarya) varieties at 10th DAF, positive trend in only five (HRC392, ISM, Mashuri, MTU 1010 and Swarnadhan) varieties at 15th DAF, positive trend in ISM alone at 20th DAF, positive trend in six (ISM, Jaya, Kalanamak, Rasi, Sugandhamati and Varadhan) varieties at 25th DAF and positive trend in two (Ganjeekalli and ISM) varieties at 30th DAF.

In amylose to amylopectin ratio in individual grain vs grain weight in mg in individual grain graph, the R^2^ value was significant for ISM and Mashuri at 10th DAF, non-significant (< 0.5) for all the varieties at 15th as well as 30th DAF’s, significant for Rasi and Tellahamsa at 20th DAF and significant for Sampada alone at 25th DAF.

### Quantification of gene expression

**Figure 6:**
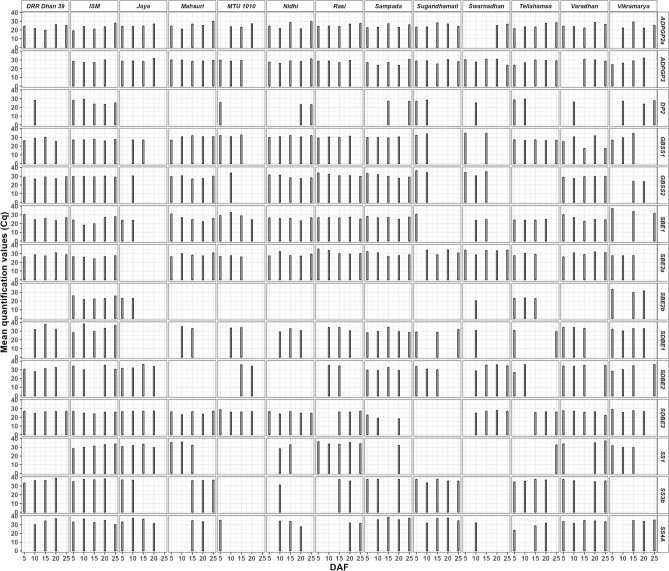
Quantitative expression of important genes involved in starch synthesis of rice varieties at various DAF’S of grain filling. ADPGP is ADP-glucose pyrophosphorylase large subunit in ADPGP2a and ADPGP3. DP is disproportionating enzyme in DP2. GBSS is granule-bound starch synthase in GBSS1 and GBSS2. SBE is starch branching enzyme in SBE1, SBE2a and SBE2b. SDBE is starch debranching enzyme in SDBE1, SDBE2 and SDBE3. SS is starch synthase in SS1, SS3b and SS4A.

In the expression analysis with fourteen reported markers of starch synthesizing enzymes (Table 5) in the thirteen varieties, the mean Cq values (Fig. 6) ranged from 17.44 for GBSS1 in Varadhan at 25th DAF to 38.89 for SS3b in DRR Dhan 39 at 20th DAF. Further, the mean Cq values varied from 21.16 to 34.58 for actin (reference marker) gene, 19.1 in ISM at 5th DAF to 30.08 in Mashuri at 25th DAF for ADPGP2a, 23.18 in Nidhi at 20th DAF to 29.69 in Tellahamsa at 10th DAF for DP2, 23.94 in Sampada at 10th DAF to 32.14 in Vikramarya at 20th DAF for ADPGP3, 17.44 in Varadhan at 25th DAF to 34.92 in Swarnadhan at 5th DAF for GBSS1, 23.68 in Vikramarya at 20th DAF to 36.21 in Sugandhamati at 5th DAF for GBSS2, 18.26 in ISM at 10th DAF to 37.32 in Vikramarya at 5th DAF for SBE1, 24.09 in ISM at 15th DAF to 35.37 in Rasi at 5th DAF for SBE2a, 20.32 in Swarnadhan at 10th DAF to 31.48 in Vikramarya at 20th DAF for SBE2b, 28.04 in Sampada at 5th DAF to 36.48 in ISM at 25th DAF for SDBE1, 27.88 in DRR Dhan 39 at 15th DAF to 36.24 in Jaya at 15th DAF for SDBE2, 18.27 in Sampada at 10th DAF to 29.05 in Vikramarya at 5th DAF for SDBE3 28.26 in Nidhi at 15th DAF to 37.34 in Varadhan at 25th DAF for SS1, 31.16 in Nidhi at 10th DAF to 38.89 in DRR Dhan 39 at 10th DAF for SS3b and 23.67 in Tellahamsa at 5th DAF to 38.49 in Sampada at 15th DAF for SS4A.

**Table 5:** List of RT-PCR primers used in gene expression analysis.

Enzyme	Gene	Accession	Forward primer	Reverse primer
ADP-glucose pyrophosphorylase large subunit 2a	OsAGPL2	U66041	AGTTCGATTCAAGACGGATAGC	CGACTTCCACAGGCAGCTTATT
ADP-glucose pyrophosphorylase large subunit 3	OsAGPL3	AK069296	AAGCCAGCCATGACCATTTG	CACACGGTAGATTCACGAGACAA
Starch synthase I	OsSSI	D16202	GGGCCTTCATGGATCAACC	CCGCTTCAAGCATCCTCATC
Starch synthase IIIb	OsSSIIIb	AF432915	ATTCCGCTCGCAAGAACTGA	CAACCGCAGGATAACGGAAA
Starch synthase IVa	OsSSIVa	AY100470	GGGAGCGGCTCAAACATAAA	CCGTGCACTGACTGCAAAAT
Granule-bound starch synthase I	OsGBSSI	X62134	AACGTGGCTGCTCCTTGAA	TTGGCAATAAGCCACACACA
Granule-bound starch synthase II	OsGBSSII	AY069940	AGGCATCGAGGGTGAGGAG	CCATCTGGCCCACATCTCTA
Starch branching enzyme I	OsBEI	D11082	TGGCCATGGAAGAGTTGGC	CAGAAGCAACTGCTCCACC
Starch branching enzyme IIa	OsBEIIa	AB023498	GCCAATGCCAGGAAGATGA	GCGCAACATAGGATGGGTTT
Starch branching enzyme IIb	OsBEIIb	D16201	ATGCTAGAGTTTGACCGC	AGTGTGATGGATCCTGCC
Starch debranching enzyme: Isoamylase I	OsISA1	AB093426	TGCTCAGCTACTCCTCCATCATC	AGGACCGCACAACTTCAACATA
Starch debranching enzyme: Isoamylase II	OsISA2	AC132483	TAGAGGTCCTCTTGGAGG	AATCAGCTTCTGAGTCACCG
Starch debranching enzyme: Isoamylase III	OsISA3	AP005574	ACAGCTTGAGACACTGGGTTGAG	GCATCAAGAGGACAACCATCTG
Disproportionating enzyme II	OsDPE2	AK067082	CAAGTACACCACAAGACCAGCAA	CGTCCAACAGCGAATCCAAT

The cumulated Cq mean values were significantly highest in eight varieties for ADPGP2a (Table 6), five varieties for ADPGP3 as well as SDBE3, seven varieties for GBSS2, two varieties for SDBE1 as well as SS4A and one variety for the remaining genes (DP2, GBSS1, SBE1, SBE2a, SBE2b, SDBE2, SS1 and SS3b). ADPGP2a was expressed at all DAF’s in all the varieties except Jaya, MTU 1010, Swarnadhan and Vikramarya. The DP2 gene was expressed at all DAF’s in ISM and after 20th DAF in Nidhi, Sampada and Vikramarya.

**Table 6:** Cumulative mean quantification values (Cq) of genes and quality parameters of the rice varieties.

Enzyme / quality parameter	DRR Dhan 39	ISM	Jaya	Mahsuri	MTU 1010	Nidhi	Rasi	Sampada	Sugandhamati	Swarnadhan	Tellahamsa	Varadhan	Vikramarya	LSD @ 0.05
ADPGP2a	116.9^a^	115.6^ab^	99.3^b^	127.3^a^	73.8^c^	125.6^a^	126.6^a^	121.7^a^	125.6^a^	51.7^d^	124.2^a^	125.4^a^	98.9^b^	16.78
ADPGP3	0.0^e^	112.7^c^	117.3^bc^	146.9^a^	87.9^d^	142.1^a^	114.0^c^	133.0^ab^	141.9^a^	143.7^a^	139.3^a^	89.2^d^	112.0^c^	14.71
DP2	28.0^d^	129.8^a^	0.0^e^	0.0^e^	25.6^d^	46.3^c^	0.0^e^	54.1^c^	55.3^c^	25.2^d^	58.3^c^	26.2^d^	78.7^b^	16.12
GBSS1	111.1^de^	135.7^bc^	54.0^ g^	151.2^ab^	95.9^ef^	155.5^a^	121.4^ cd^	119.8^ cd^	66.5^ g^	69.7^ g^	134.3^c^	122.5^ cd^	91.1f.	16.8
GBSS2	141.2^a^	147.5^a^	30.2^e^	143.8^a^	33.7^de^	146.2^a^	156.6^a^	151.4^a^	70.3^c^	99.9^b^	0.0f.	145.0^a^	47.6^d^	16.41
SBE1	130.8^abc^	117.3^bcd^	47.7	130.7^abc^	115.4^ cd^	128.3^abc^	132.8^ab^	134.6^a^	30.8^ g^	48.3f.	96.7^e^	128.9^abc^	102.9^de^	16.78
SBE2a	142.3^cde^	130.7^de^	0.0^ g^	143.1^bcde^	80.7f.	144.2^bcd^	158.7^ab^	146.4^bcd^	127.6^e^	163.3^a^	87.1f.	149.3^abc^	82.8f.	16.13
SBE2b	0.0f.	119.0^a^	46.3^d^	0.0f.	0.0f.	0.0f.	0.0f.	0.0f.	0.0f.	20.3^e^	69.4^c^	0.0f.	94.9^b^	10.41
SDBE1	101.0^c^	165.5^a^	0.0f.	68.0^d^	67.4^d^	92.4^c^	98.6^c^	149.9^a^	89.3^c^	30.7^d^	60.2^d^	101.4^c^	128.0^b^	16.15
SDBE2	122.8^b^	129.9^ab^	134.0^ab^	0.0^e^	69.9^d^	0.0^e^	69.4^d^	120.7^b^	94.6^c^	134.5^ab^	63.0^d^	138.7^a^	129.2^ab^	15.43
SDBE3	130.7^a^	127.2^a^	107.0	126.3^a^	107.2^b^	125.9^a^	78.7^c^	59.8^d^	0.0^e^	106.5^b^	77.6^c^	128.5^a^	108.6^b^	16.76
SS1	0.0^ h^	156.6^b^	126.0^c^	104.0^de^	0.0^ h^	61.2f.	173.2^a^	32.2^ g^	0.0^ h^	0.0^ h^	32.7^ g^	106.4^d^	91.4^e^	13.96
SS3b	144.8^b^	147.9^b^	73.8^d^	109.2^c^	0.0f.	31.2^e^	73.1^d^	112.6^c^	180.0^a^	0.0f.	144.7^b^	144.3^b^	0.0f.	14.71
SS4A	101.4^c^	167.3^a^	138.2^b^	68.2^ef^	35.2^ g^	95.4^ cd^	63.6f.	147.3^b^	141.2^b^	32.2^ g^	84.2^de^	167.8^a^	103.9^c^	16.78
ASV	5.0^b^	4.0^c^	7.0^a^	5.0^b^	4.0^c^	5.0^b^	7.0^a^	5.0^b^	7.0^a^	7.0^a^	7.0^a^	4.0^c^	7.0^a^	0.86
AC	20.8^b^	17.1^d^	16.0^e^	13.5f.	16.8^d^	13.9	21.9^a^	14.5f.	18.3^c^	15.6^e^	16.6^d^	20.7^b^	15.4^e^	1.82
AP	51.7f.	67.9^b^	68.5^b^	72.1^a^	57.2^d^	55.8^e^	64.7^c^	36.9^ g^	58.6^d^	64.0^c^	68.8^b^	64.3^c^	63.9^c^	5.32
GC	47.0^d^	22.0^ g^	22.0^ g^	22.0^ g^	59.0^b^	42.0^e^	22.0^ g^	54.0^c^	22.0	25.0f.	22.0^ g^	62.0^a^	23.0^ g^	4.48

Different letters after each data point indicate statistically significant differences at *p* < 0.05. Data point with no common letters are significantly different (*p* < 0.05).

Expression was not observed for ADPGP3 in DRR Dhan 39, GBSS2 in Tellahamsa, DP2 in three (Jaya, Mashuri and Rasi) varieties, SBE2a in Jaya, SBE2b in eight varieties that showed medium (> 40 mm) to soft (> 60 mm) gel consistency (Table 6), SDBE1 in Jaya, SDBE2 in Mashuri, SDBE3 in Sugandhamati, SS1 in four varieties and SS3b in three varieties.

**Figure 7:**
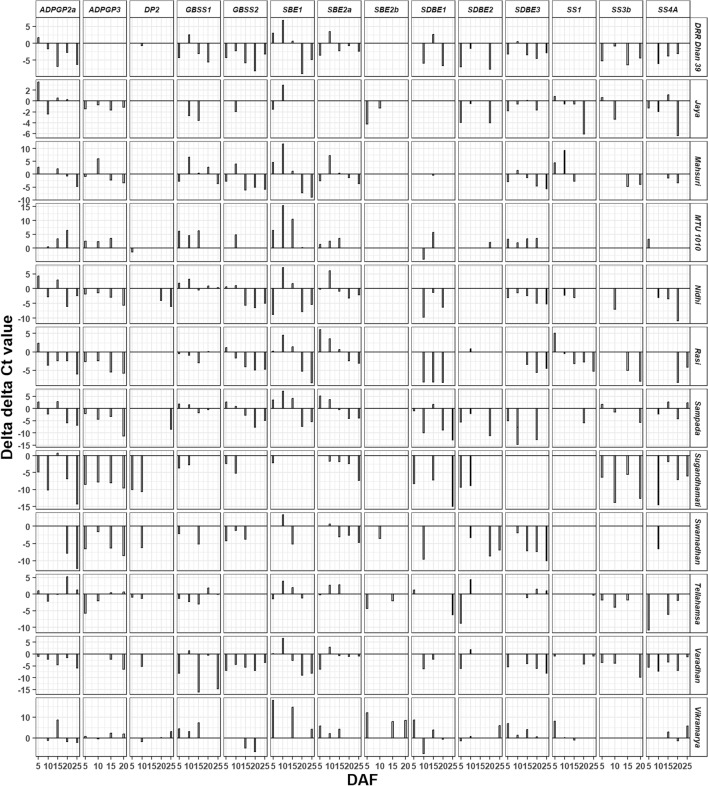
Delta delta Ct values of starch synthesizing genes in comparison with ISM.

Compared with ISM, the ∆∆Ct (ddCt) values or the fold change of all the fourteen genes at all DAF’s were downregulated in Sugandhamati and in most of the other varieties. Whereas these values were upregulated in MTU 1010 except SDBE gene (Fig. 7). Highest upregulation values (> 10) were observed for SBE1 as well as SBE2b genes in Vikramarya and for SBE1 gene in MTU1010. Highest downregulation values (< -10) were observed in most of the varieties except Jaya and Mashuri.

### Relation between gene expression and grain cooking quality

**Figure 8:**
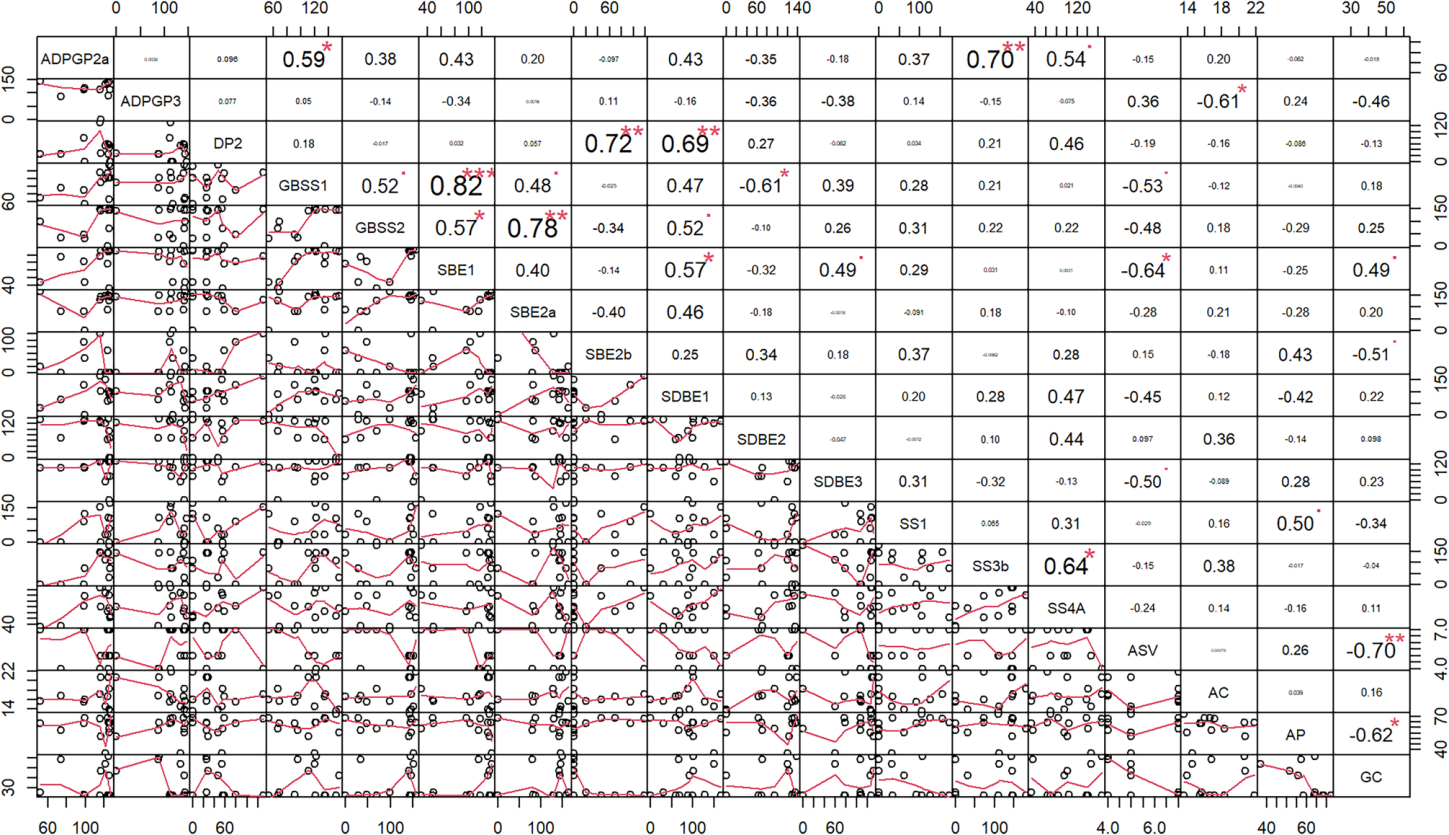
Pearson correlation chart among the cumulative Cq mean of starch synthesizing genes and grain quality parameters. The x and y axis represent the two parameters that are being correlated respectively. In the case of GC vs AP (the correlation value is -0.62*), GC is x axis while AP is Y axis. ADPGP is ADP-glucose pyrophosphorylase large subunit in ADPGP2a and ADPGP3. DP is disproportionating enzyme in DP2. GBSS is granule-bound starch synthase in GBSS1 and GBSS2. SBE is starch branching enzyme in SBE1, SBE2a and SBE2b. SDBE is starch debranching enzyme in SDBE1, SDBE2 and SDBE3. SS is starch synthase in SS1, SS3b and SS4A. ASV is alkali spreading value. AC is amylose content. AP is amylopectin content. GC is gel consistency.

In multiple correlation analysis (Fig. 8), alkali spreading value showed significant (P 0.05) negative correlation with SBE1 followed by GBSS1 and SDBE3. Amylose content showed significant (P 0.05) negative correlation with ADPGP3. Amylopectin content showed significant (P 0.05) positive correlation with SS1. Gel consistency noted significant negative correlation with alkali spreading value (P 0.01), amylopectin content and SBE2b (P 0.05) while it noted significant positive correlation with SBE1 (P 0.05). ADPGP2 showed significant positive correlation with GBSS1 (P 0.05), SS3b (P 0.01) and SS4a (P 0.05). DP2 showed significant (P 0.01) positive association with SBE2b and SDBE1.

## Discussion

Grain filling is one of the important stages of the rice crop that can influence both yield and quality. In general, the duration of grain filling is 30 days from the day of pollination. Before attaining physiological maturity, grains in the panicle are at various growth stages. Starch is the main component of rice grain that includes amylose and amylopectin. Amylose content is the chief parameter in selecting good quality rice varieties. However, varieties with similar amylose content show variation in other quality parameters. Hence, at various DAF’s, the quantities of amylose and amylopectin were estimated in relation to the grain weight in the varieties varying in yield and the varieties having similar amylose content. In addition, gas exchange parameters were quantified in varieties varying in yield. Expression of fourteen genes was quantified in varieties that contain similar amylose content.

### Gas exchange parameters

**Figure 9:**
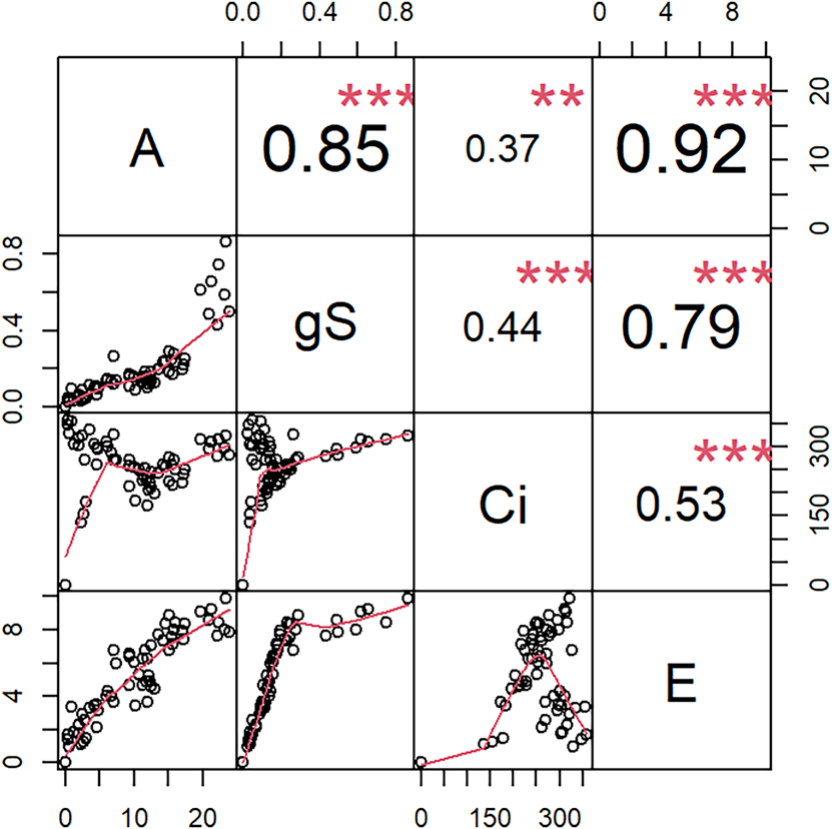
Pearson correlation chart among the gas exchange parameters. The x and y axis represent the two parameters that are being correlated respectively. A: photosynthetic rate, gS : stomatal conductance, Ci : intercellular CO_2_ and E: transpiration rate.

Most of the values of gas exchange parameters decreased from the 5th to 30th DAF during grain filling. Like^31,32^, compared with poor yielders, the flag leaves of good yielders could retain photosynthetic activity for more DAF’s during the grain‐filling period. Photosynthetic rate (A) is a function of intercellular CO_2_ (Ci) which in turn is a function of stomatal conductance (g_s_)_._ Like^33,34^, A showed significant correlation with g_s_ and E (Fig. 9). Grain yield can be influenced by A, gs and area as well as shape of the flag leaf ^35–37^. Increase in A will increase canopy carbon gain, accumulation of dry matter at late growth stages leading to increased yield and crop biomass^34,38^. The present results emphasize to record the gas exchange parameters around 15th to 20 DAF’s in addition to the popular practice of taking measurements after reaching full vegetative stage to delineate varieties based on yield.

### Panicle topology and grain weight

In each branch, during grain filling, grains at various growth stages (individual grain weights) were observed (Fig. 1). Simultaneously, multiple spikelets acted as sink irrespective of their position in the panicle (branch or sub-branches). In Kalanamak, sub-branches were noticed in each branch. In general, the thickness or diameter decreased from the main rachis to the branches, respective sub-branches (if present) and in each branch or sub-branch from their point of origin to the tips. This enables the main rachis to contain more amount of sap that can be distributed to multiple branches simultaneously. Within a branch or sub-branch, the flowers that underwent pollination earlier than other flowers might be acting as independent sinks and not necessarily the grains at the tip of the branch or in the top sub-branch. If filling is only from top to bottom, grain filling cannot initiate in multiple branches (Fig. 1). Hence, panicle collected at 10th, 15th or 20th DAF can represent various developmental stages despite the total number of partially or completely filled grains increased from 10 to 30th DAF (Table 4). The mean and maximum dry weights of the individual grains in the panicle were similar from 15 to 30th DAF in most of the varieties. Further, the individual grains attain the characteristic (maximum) grain weight of the genotype either before or at the physiological maturity.

Each rice variety also contains a specific grain type which can be short slender, short bold, medium slender, medium bold, long slender or long bold. Generally, the individual grain weight is least for short slender and highest for long bold grain type. Hence, the final grain yield relies on the panicle number, grain number and grain type. Of the four varieties studied for yield, lower characteristic individual grain dry weight (10 mg) can be one of the reasons for the poor yield of Kalanamak (medium slender) despite having more total grain number (Table 4). Except in two varieties (Ganjeekalli and HRC392), the minimum individual grain weight values were significantly lower than the maximum individual grain weights even at 30th DAF (Table 4). The maximum individual dry grain weight was higher in superior (24 mg in Agnisera and 21 mg in Swarna) than inferior (13 mg in Agnisera and 7 mg in Swarna) grains^18^.

### Relation between starch components and grain weight

Compared with amylose, amylopectin being a branched molecule (availability of many 4-OH ends), absorbs glucose at a faster rate. The observed negative trend for amylopectin (Figs. 3 and 5) and positive trend for amylose formation (Figs. 2 and 4) in most of the varieties indicates that the absorption of sugar and it’s conversion to starch can be higher in the early days of their growth. Further, the highly significant or significant R^2^ value in most of the varieties (Supplementary Figs. 1 and 2) indicates that the accumulated quantities of amylose and amylopectin in the individual grain can be similar in all the grains having similar grain weight. Unlike the current results, although differential quantities of maximum starch content were reported from superior (12 mg in Agnisera and 7 mg in Swarna) and inferior (6 mg in Agnisera and 3 mg in Swarna) grains^18^, the starch contents were analyzed by pooling grains from seven plants in each replicate rather in individual grain separately.

As mentioned in the introduction, even in grain filling studies, amylose and amylopectin were estimated by various methods using rice powders (made from multiple grains) ^30,31^. The popular amylose estimation method^5^ measures the colour of iodine-amylose complex at 620 nm (single wavelength), where, the colour of iodine-amylopectin complex also shows significant absorption leading to higher amylose content (4.04% for a standard mixture having 37.7% amylopectin and 0% amylose)^10^. Similarly, higher amylose content of 16.48% was obtained for a standard mixture having 75% amylopectin and 0% amylose^39^. As rice grain contains both amylose and amylopectin, usage of single wavelength based amylose estimation method can give higher values. As rice grain with a specific grain weight during various intervals of grain filling can represent a specific growth stage, a previously standardized in-house method (Sanjeeva Rao and Subrahmanyam, IRS, 2015, IRS15-PP 370) that enables to disperse individual grain into a solution was employed in this study. An aliquot of the solution was treated with iodine solution to quantify the amylose and amylopectin through non-interference equations^10^.

### Quantification of gene expression

ADPGP2a and ADPGP3 are isoforms and perform the same function^40^. Hence, absence of ADPGP2a in four varieties (Jaya, MTU 1010, Swarnadhan and Vikramarya) may not show significant effect on starch synthesis.

Expression of GBSSI was observed at all or few DAF’s in the varieties (Fig. 6). Although GBSSI is responsible for the synthesis of amylose, non-significant correlation was noted between GBSSI and amylose content (Fig. 8). So far, 10 Wx natural alleles that differ in sequence as well as expression leading to variation in amylose content were identified in rice^41^. It includes the novel Wx allele (devoid of 1^st^ intron leading to 1–2 folds enhanced mRNA as well as amylose content) which was knocked out to obtain glutinous or sticky rice ^42^. Wxa and Wxb are the two major alleles that are distributed widely in most *indica* and *japonica* rice varieties that contain high and low amylose respectively.

The disproportioning enzyme’s- DP1 and 2 were considered to participate in starch degradation and their expression was expected in the initial days of grain filling^43^. DP1 transfers maltose unit from one maltotriose (G3) to another maltotriose (G3) to synthesize maltopentose (G5) or transfers maltose unit from G7 to glucose to form G3. Whereas, DP2 adds glucose from maltose to amylopectin^43^. Although, change in the expression of during grain filling can alter the size, morphology and structure of the starch granule^44^, DP1 noted non-significant correlation with all the four (Fig. 8) cooking quality parameters (ASV, GC, AC and AP).

Starch branching enzyme (SBE) alone can introduce branches through α-1,6-glycosidic bonds in the linear molecule to convert it into a branched molecule (amylopectin). Rice contains two isoforms of SBE- SBEI and SBEII. SBEII inturn contains two isoforms- SBEIIa and SBEIIb.

SBEI transfers longer chains that link multiple clusters of amylopectin with the medium size chains in the amylopectin amorphous lamella. It’s deficiency can decrease long chains with DP ≥ 37 and short chains with 12 ≤ DP ≤ 21 and simultaneously, increase short chains with DP ≤ 10 and 24 ≤ DP ≤ 34^45^.

SBEIIa is present in vegetative tissues and is absent in the endosperm^46^. However, in this study, its expression was observed in the rice grain of all the varieties except Jaya. It incorporates DP11-22 (intermediate chains) of amylopectin and compensates BEI than BEIIb^47^. BEIIb is expressed exclusively in the endosperm, BE2b mutants contain less quantity of DP ≤ 14 chains in amylopectin and lead to a drastic difference in endosperm starch structure compared with the wild-type japonica rice^48^. Similar changes in amylopectin were observed in BEIIb CRISPR/Cas9 mutant of *indica* rice which has higher number of branches with DP 6, 7 and > 25 and lower number of chains with DP 9–24 leading to increased gelatinization temperature by 6 °C and increased amylose content by 7%^49^. Absence of BEIIb decreased the short chain number and increased the long chain number simultaneously^50^. In this study, SBEI expression was noted in all the varieties. Although SBEI expression was observed at one DAF in Sugandhamati, it’s gel consistency was less and alkali spreading value was high due to the presence of SBEIIa which can compensate SBEI^47^. The eight varieties that lack SBE2b can be devoid of short chains in the amylopectin and five of them showed medium to soft gel consistency (Table 6, Fig. 8).

Starch synthase SSI extends the DP4-7 short chains of amylopectin to DP 7-11. SS4A is expressed in all parts of the plant including developing grains and may be involved in the priming of the starch granule. However, it’s absence can be compensated by SS3^51^. SSII and SSIII synthesize medium and long amylopectin chains respectively. As the expression of SS4A was noticed in all the 12 varieties, the absence of SS3b in MTU1010, Swarnadhan and Vikramarya may not affect the priming of starch synthesis.

Allelic variations of SS2a gene change the viscosity and gelatinization temperature (alkali spreading value) of rice starch. It elongates the branches of amylopectin in endosperm^52^. Interaction among these genes can create rice varieties with novel grain quality and various combinations of available cum deficient alleles (GBSSI, SS2a, SS3a, SS4b, ISA1 and BE2b) showed unique starch properties such as increasing resistant starch for improving human health benefits^50,53^.

SDBE or isoamylase removes the improperly positioned branches and imparts crystallinity in the starch granule^28^. SDBE1 is important for amylopectin formation and its absence forms randomly branched water soluble amylopectin^54^. Its function is similar to SDBE2 and SDBE3 and forms the wild type starch^55,56^. Therefore, absence of SDBE1 in Jaya, SDBE2 in Mashuri and SDBE3 in Sugandhamati may not affect the starch structure since other two isoforms are present.

The significant positive association or correlation between the expression of ADPGP2a with GBSS1, SS3b and SS4A indicates that the rate of activation of glucose matches with the accumulation of amylose by GBSS1 and long chains of amylopectin by SS3b and SS4A. Similarly, the significant positive association between GBSS1 with SBEI and SBE2a indicates the utilization of linear chains synthesized by GBSS1 by SBEI and SBE2a to incorporate longer and intermediate branches in the starch components. The significant positive association between DP1 with SBE2b and SDBE1 indicates that their concerted action in introducing smaller branches in the amylopectin.

Biomolecules like mRNA and protein (enzyme) also possess half-life which can vary among the genotypes. Further, the efficiency of translation of the mRNA and activity of the enzyme may also vary in the genotypes. In other words, presence of gene expression at a DAF and its absence in the subsequent DAF may not suggest the absence of the functional protein or enzyme activity. Hence, the genotypes employed for gene expression study possess similar amylose content despite the variation in the expression of GBSS1 and GBSS2.

However, rice varieties having similar or same amylose content can differ in the quantity as well as structure of amylopectin due to variation in the availability or expression of other enzymes involved in starch synthesis.

### Relation between gene expression and grain cooking quality

Alkali spreading value ranges from a scale of 1 (no change) to 7 (complete dispersion of grain) upon submersion of intact milled or polished rice grains in alkali for 23 h^4^. Alkali spreading value is indirectly proportional to starch gelatinization temperature while cooking in water. It’s significant negative association with GBSS1, SBE1 and SDBE3 indicates that presence of amylose, longer chains in amylopectin and or better reorganization of starch granule can increase the temperature to gelatinize the starch.

Gel consistency^6^ values of rice powder cooked in the presence of alkali range from 22 (hard or highly viscous) to 100 mm (soft or less viscous). In India, rice having high amylose (> 25%) is desirable if it contains soft (> 60 mm) gel consistency. It’s significant positive association with SBE1 indicates longer branches in amylopectin can decrease the viscosity of the gels of the cooked rice powder. It’s significant negative association with alkali spreading value again supports the negative association between alkali spreading value and SBE1 (Fig. 8). The genotypes devoid of SBE2b can contain a greater number of longer branches^48^ leading to decrease in the viscosity of the rice powders. Interestingly, increase in amylopectin can decrease the viscosity of the rice powders since amylopectin contains lesser number of cross linkages (hydrogen bonding) being a branched molecule ^57^.

## Conclusions

The photosynthetic activity lasted for longer time during grain filling period in good yielders. In most of the varieties, percentage of amylopectin was higher in the initial days of grain filling where grain weight was lesser. The quantities of amylose as well as amylopectin increased with the increase in grain weight during grain filling. The maximum amount of amylose, amylopectin and individual grain weight is characteristic or specific to a variety. To the best of our knowledge, this is the first report on the relation of amylose and amylopectin contents with the individual grain weights during grain filling. As varieties having similar amylose content (%) differed in amylopectin content (%), gel consistency and alkali spreading value, gene expression of fourteen genes was quantified using fourteen gene-specific reported primers. Of these, absence of SBE2b can be responsible for medium or soft gel consistency in five varieties having similar amylose content.

## Materials and methods

This study was conducted in two wet seasons (2018 and 2019) in the experimental fields of ICAR-Indian Institute of Rice Research, Hyderabad. In 2018, four varieties (Table 1) that differ in yield (two good yielders- Ganjeekalli and Rasi and two poor yielders- HRC392 and Kalanamak) were studied. In 2019, 12 varieties (Table 2) that contain similar amylose content including Improved Samba Mashuri (ISM) were studied. ISM was developed in the background of Samba Mashuri which is popular for best cooking quality. Rice varieties were cultivated in field following standard package of practices.

The seeds were sown on nursery beds and 25 days old seedlings were transplanted as single seedling per hill in the field a uniform spacing of 20 cm between rows and 15 cm between plants. A layer of 2–3 cm water was maintained constantly till the establishment of seedlings. Thereafter about 5 cm of water was maintained upto dough stage of the crop. The farm soil is alkaline vertisol, with a pH of 7.94. The recommended dose of fertilizers 100 kg ha^−1^ of Nitrogen in three split doses with one fourth as basal dose, one half at the time of initial tillering stage and one fourth at active tillering stage, entire dose of 40 kg ha^−1^ of phosphorus and 60 kg ha^−1^ of Muriate of Potash (MOP) per hectare were applied at the time of transplanting as basal dose. Required weed management and plant protection measures were timely undertaken for healthy crop production.

### Collection of panicles

In both the wet seasons, panicles of each variety were tagged with labels with date of anthesis (DAF). Panicles of each variety were separately collected at every five-day interval (5th, 10th, 15th, 20th, 25th and 30th) for a period of 30 days. Individual panicles were kept in respective brown cover, dried under shade and stored under ambient conditions till further analysis. Further, in wet season 2019, the top two branches of three panicles of 13 (12 + Rasi) varieties were also collected during the above intervals into RNA later and were immediately stored in a deep freezer maintained at − 20 °C till further analysis.

### Gas exchange

In 2018, on the day of panicle collection from the four varieties, gas exchange was measured in the respective flag leaf using a LI6400XT portable photosynthesis measuring system (LI-COR Environmental, USA), connected to a leaf chamber fluorimeter 6400-40 (LI-COR, USA), which was used as light source. All measurements were taken at mid-day (10:00 am to 13:00 pm). During photosynthesis measurements, leaf temperature was maintained at 35 ºC, which was similar with the ambient temperature and photosynthetic photon flux density was maintained at 1200 μmol/(m^2^ s). The measurements were made at ambient CO_2_ level (387 ± 1.2 μmol/mol) and flow rate of air was maintained at 300 μmol/s as per the manufacturer’s instructions. Measurements were taken on three replicates per variety on three different plants.

### Panicle topology

Branches of each panicle were numbered from bottom (least number) to the top (highest number). Spikelet or growing grain of each branch were carefully separated from each branch and counted. All the partially filled or completely filled grains of branch were kept in small butter paper covers that were labelled with the branch number. All the packets of each panicle were kept in the brown cover and stored under ambient conditions. In addition, in 2018, length of the panicles as well as the branches was also measured.

### Estimation of grain weight

At the end of three months, the above covers were kept for 72 h in an incubator maintained at 50 °C to remove the moisture. After incubation, the covers were transferred into a desiccator having dried silica-gel crystals to cool the samples to room temperature. Each partially filled or completely filled grain from each branch was transferred into a pre-labelled and dry 15 ml graduated tube having screw-cap lid and the individual grain weight was noted using a digital weighing balance.

### Grain quality analysis

All the individual grains collected from all the panicles of the four varieties during wet season 2018 were analysed for amylose and amylopectin as given below. One ml of 1N NaOH solution was added into each pre-labelled and dry 15 ml graduated tube that contains individual grain and all the tubes were incubated overnight where the contents of the grain was released or dispersed (Sanjeeva Rao and Subrahmanyam, IRS, 2015, IRS15-PP 370). At the end of incubation, all the tubes were kept in boiling water for 15 min, cooled, diluted with distilled water upto the 10 ml mark and the contents were mixed uniformly. An aliquot of this solution was taken into another test-tube, the contents were acidified by adding 0.1 ml of 1N glacial acetic acid followed by the addition of 0.2 ml of iodine reagent and the total volume was made upto 10 ml by adding distilled water ^5^. The tubes were incubated for 20 min at room temperature in dark and the intensity of the colour was measured at two wavelengths 560 nm (λmax of amylopectin-iodine complex) and 620 nm (λmax of amylose-iodine complex). The absorbance values were substituted in the non-interference equations^10^ to derive the amylose and amylopectin values without the interference of amylopectin in amylose ^9^ and vice-versa. Based on these results, in 2019, three grains representing similar grain weight (from least to highest grain weights of the panicle) of all varieties at various DAF’s were analysed.

Further, the harvested seed after physiological maturation were dried under shade to reach 14% moisture and stored under ambient conditions. At the age of three months, paddy was dehusked to brown rice which was milled to obtain polished rice. About one g of polished grains of each variety were manually ground into powders (100 mesh) in a mortar and pestle. Polished grains were subjected to alkali spreading value and rice powders were subjected to amylose content, amylopectin content and gel consistency ^4–6^ analysis.

### Gene expression analysis

Five grains from the top two branches of the panicle (50–100 mg) were used to isolate total RNA by Trizol method ^58^. The extracts were treated with RNase-free DNase to completely remove the genomic DNA. The purity and concentration of diluted RNA sample was estimated by measuring absorbance at 260 nm and 280 nm using a NanoDrop ^59^. RNA was used as the template for first-strand cDNA synthesis with the prime script cDNA synthesis kit (TAKARA) ^60^. Aliquots of the first-strand cDNA mixtures served as the templates for quantitative real-time PCR analysis in Light Cycler (Roche) according to the manufacturer’s protocols and the gene-specific primers (Table 5). All the reactions were run in triplicate with actin as an internal control. The mean quantification values (Cq) values were further converted to ∆Ct values by removing the mean Cq value of actin from the mean Cq value of each gene in a genotype. Finally, ∆∆Ct values were derived using ISM as reference genotype ^61^.

### Statistical analysis

All the graphs were developed in R software using various packages as mentioned below. The single grain weight in individual branches of the panicle and regression graphs of amylose and amylopectin with increase in single grain weight at various DAF’s were developed using ggplot2 and ggpmisc packages. The mean quantification values (Cq) and ∆∆Ct were used to develop graphs using ggplot2 package and multiple correlation analysis along with grain quality parameters using performance analytics package.

All the experiments carried out on plants were carried out in accordance with the guidelines of Indian Council of Agricultural Research (ICAR)—Indian Institute of Rice Research (IIRR).

### Supplementary Information

Supplementary Figures.

## Data Availability

The datasets used and/or analysed during the current study are available from the corresponding author on reasonable request. The cultivation of plants and collection of plant samples were done following the institutional procedures. Gas exchange parameters, grain quality analysis were performed following peer reviewed published literature and were cited appropriately. Reported primers were used to study gene expression.
