# Supplementary material for: Dynamics of starch formation and gene expression during grain filling and its possible influence on grain quality
Source: Sci Rep. 2024 Mar 21;14:6743. doi: 10.1038/s41598-024-57010-4 (PMC10954615; doi:10.1038/s41598-024-57010-4)
Supplement: Supplementary file 1 — Supplementary Figures. [file 41598_2024_57010_MOESM1_ESM.pdf]

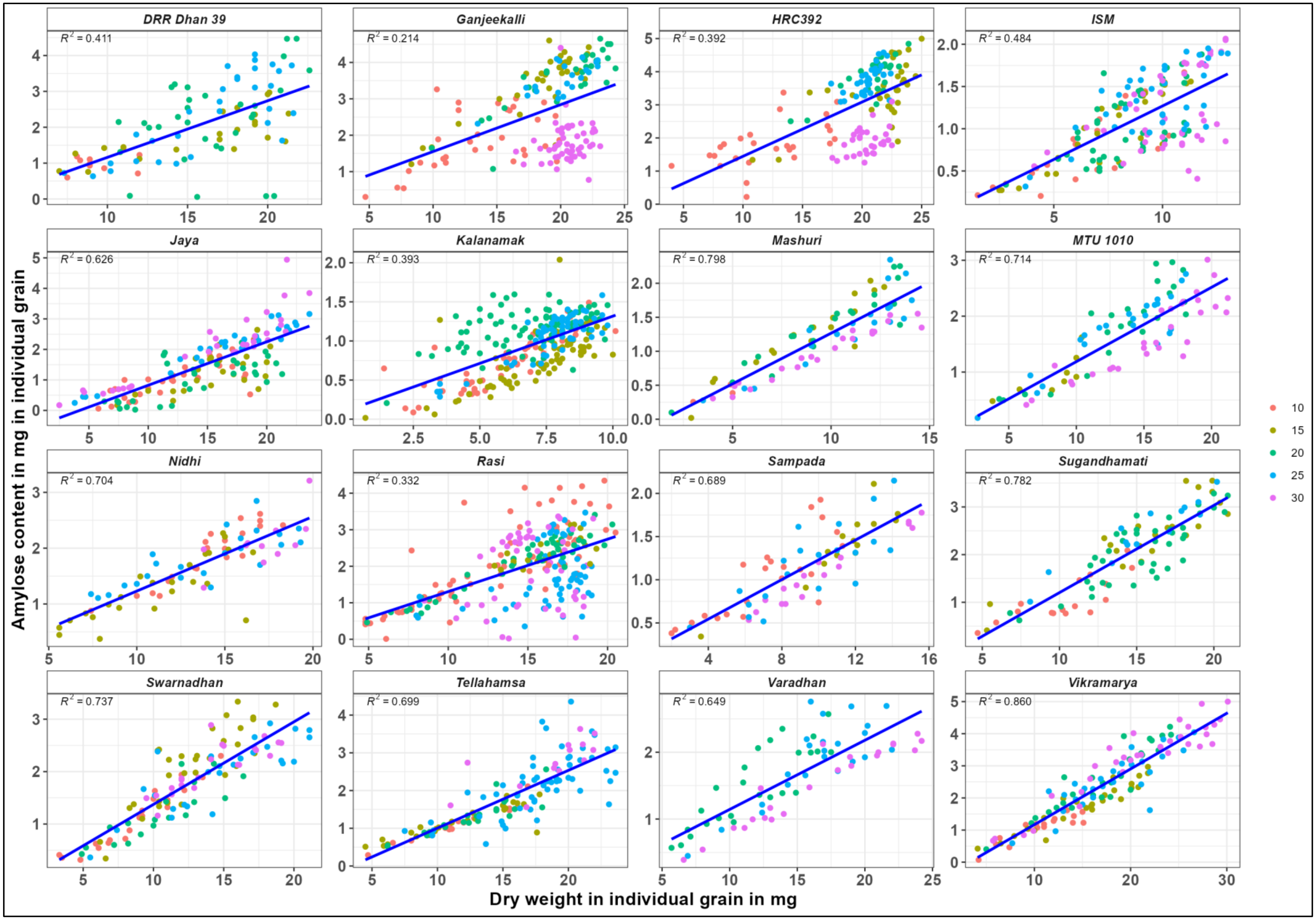

Supplementary Figure 1. Amylose content in mg in individual grains of rice varieties during grain filling.

10 – 10th day after flowering; 15 - 15th day after flowering; 20 - 20th day after flowering; 25 - 25th day after flowering; 30 - 30th day after flowering

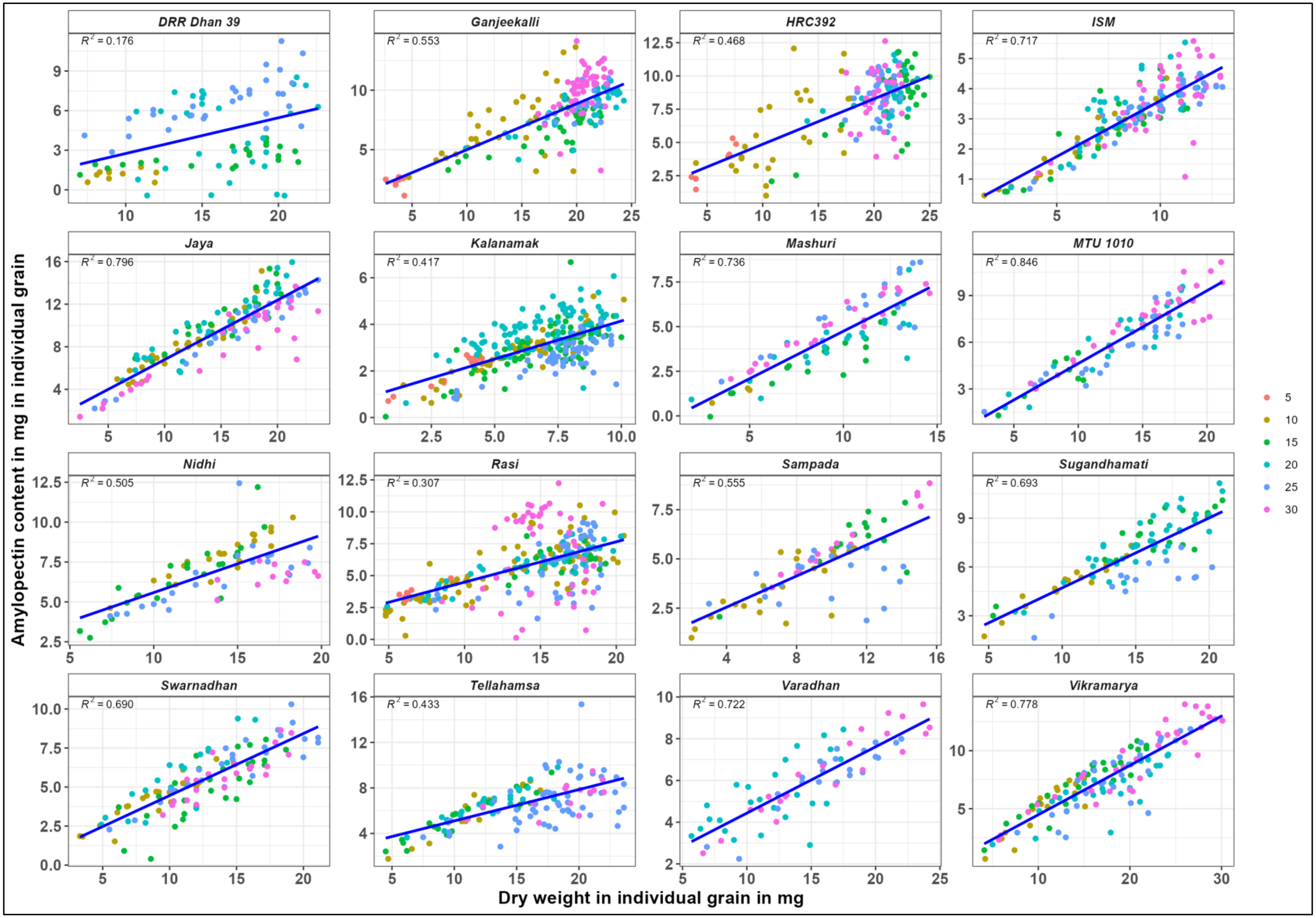

Supplementary Figure 2. Amylopectin content in mg in individual grains of rice varieties during grain filling.

10 – 10th day after flowering; 15 - 15th day after flowering; 20 - 20th day after flowering; 25 - 25th day after flowering; 30 - 30th day after flowering

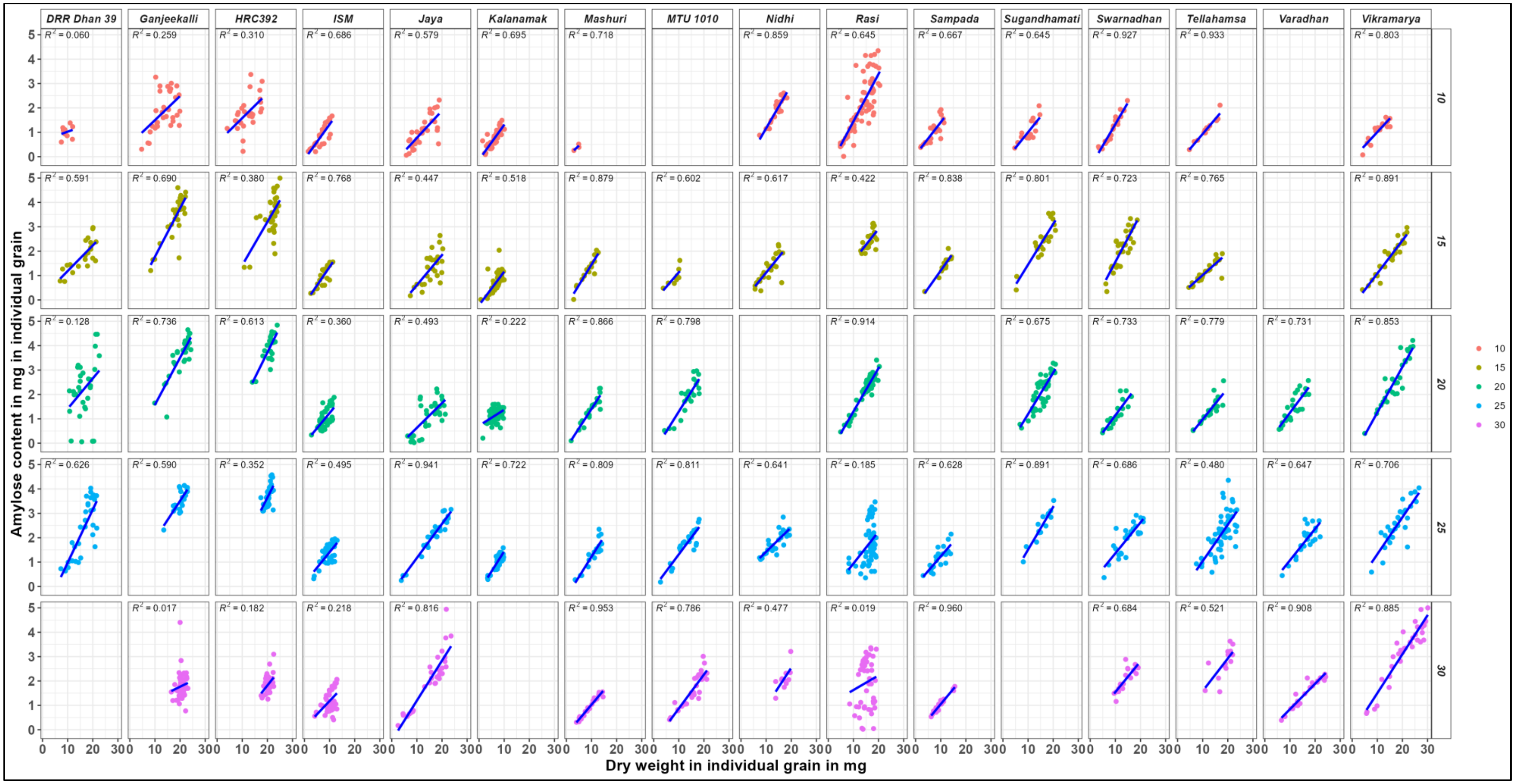

Supplementary Figure 3. Amylose content in mg in individual grains of rice varieties during grain filling.

Regression line trend was shown at each DAF in individual genotypes. 10 – 10th day after flowering; 15 - 15th day after flowering; 20 - 20th day after flowering; 25 - 25th day after flowering; 30 - 30th day after flowering

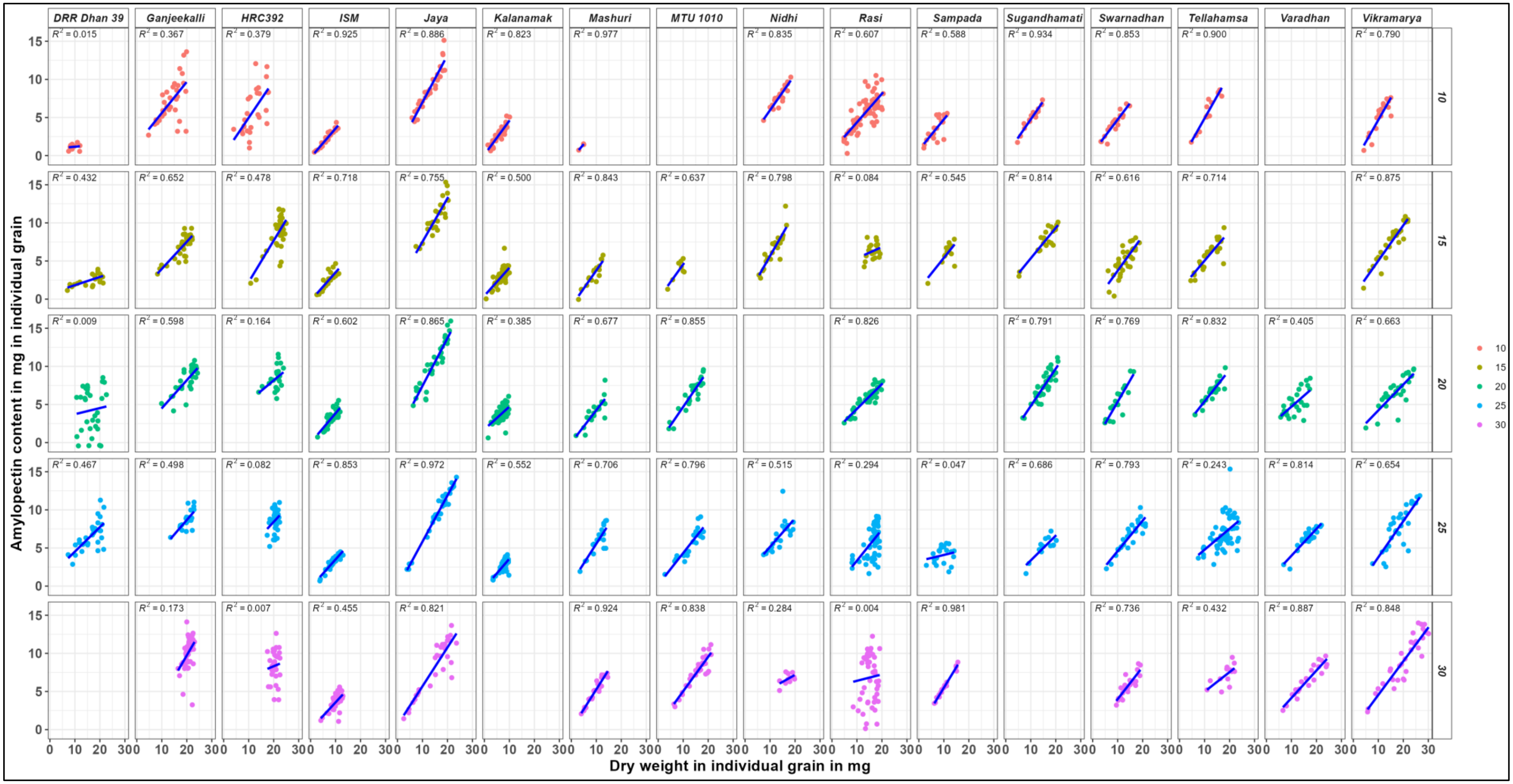

Supplementary Figure 4. Amylopectin content in mg in individual grains of rice varieties during grain filling.

Regression line trend was shown at each DAF in individual genotypes. 10 – 10th day after flowering; 15 - 15th day after flowering; 20 - 20th day after flowering; 25 - 25th day after flowering; 30 - 30th day after flowering
